# Enzymatic Synthesis of *N*-Acetyllactosamine (LacNAc) Type 1 Oligomers and Characterization as Multivalent Galectin Ligands

**DOI:** 10.3390/molecules22081320

**Published:** 2017-08-10

**Authors:** Thomas Fischöder, Dominic Laaf, Carina Dey, Lothar Elling

**Affiliations:** Laboratory for Biomaterials, Institute for Biotechnology and Helmholtz-Institute for Biomedical Engineering, RWTH Aachen University, Pauwelsstraße 20, 52074 Aachen, Germany; t.fischoeder@biotec.rwth-aachen.de (T.F.); d.laaf@biotec.rwth-aachen.de (D.L.); c.dey@biotec.rwth-aachen.de (C.D.)

**Keywords:** neo-glycoproteins, biocatalysis, LacNAc type 1, chemo-enzymatic synthesis, one-pot, sequential, glycosyltransferase, galectin-3, multivalency

## Abstract

Repeats of the disaccharide unit *N*-acetyllactosamine (LacNAc) occur as type 1 (Galβ1, 3GlcNAc) and type 2 (Galβ1, 4GlcNAc) glycosylation motifs on glycoproteins and glycolipids. The LacNAc motif acts as binding ligand for lectins and is involved in many biological recognition events. To the best of our knowledge, we present, for the first time, the synthesis of LacNAc type 1 oligomers using recombinant β1,3-galactosyltransferase from *Escherichia coli* and β1,3-*N*-acetylglucosaminyltranferase from *Helicobacter pylori*. Tetrasaccharide glycans presenting LacNAc type 1 repeats or LacNAc type 1 at the reducing or non-reducing end, respectively, were conjugated to bovine serum albumin as a protein scaffold by squarate linker chemistry. The resulting multivalent LacNAc type 1 presenting neo-glycoproteins were further studied for specific binding of the tumor-associated human galectin 3 (Gal-3) and its truncated counterpart Gal-3∆ in an enzyme-linked lectin assay (ELLA). We observed a significantly increased affinity of Gal-3∆ towards the multivalent neo-glycoprotein presenting LacNAc type 1 repeating units. This is the first evidence for differences in glycan selectivity of Gal-3∆ and Gal-3 and may be further utilized for tracing Gal-3∆ during tumor progression and therapy.

## 1. Introduction

The *N*-acetyllactosamine type 1 (LacNAc type 1, Galβ1,3GlcNAc) is a well-known precursor of several important blood group epitopes, such as Lewis A, Lewis B, or sialyl Lewis A [1], which are involved in many biological processes, e.g., fertilization [2] and pathogen adhesion [3]. Furthermore, LacNAc type 1 moieties, as well as Lewis type epitopes also occur in human milk [4,5,6]. The LacNAc type 1 glycosylation motif itself, as part of Lewis epitope structures [7] or in repetitive sequences [8,9], has been frequently found in gastrointestinal tissues [8,10,11,12,13], lung [14] or urothelium [15]. LacNAc type 1 containing glycans play also an important role in tumor metastasis [7] and are, therefore, considered as tumor markers [13]. Repetitive sequences, i.e., dimeric LacNAc type 1 structures (di-LacNAc type 1) were identified on lactosylceramides from colonic adenocarcinoma and found in colon cancer cell lines [8,9]. We conclude that there is a rising need for the synthesis of glycoconjugates containing LacNAc type 1 repeats to gain novel insights into the interaction of carbohydrates and carbohydrate binding proteins, known as lectins. Recently, we reported on the synthesis of LacNAc type 1 oligomers consisting of up to four repetitive LacNAc units by combining a glycosyltransferase and an engineered glycosynthase [16]. However, the synthesis required the chemically synthesized α-galactopyranosyl fluoride (αGalF) as specific donor substrate, which is prone to hydrolysis. An alternative synthesis approach is the use of a novel β1,3-galactosyltransferase from *E. coli* O55:H7, which belongs to the *Leloir*-glycosyltransferase family 2 (GT2, EC 2.4.1.-) and utilizes uridine 5′-diphosphate (UDP-)α-d-galactose (UDP-Gal) as glycosyl donor (termed WbgO in reference [17]). The availability of UDP-Gal and other nucleotide sugars was significantly improved by optimized in vitro syntheses during the last years [18,19].

Galectins are lectins which preferably bind β-galactoside glycan structures. Due to their dimeric or multimeric character, galectins mediate cellular communication events by glycan crosslinking [20,21,22,23,24,25]. In this way, galectins trigger immune responses and cancer progression [26,27,28,29,30,31,32]. Among the fifteen human galectins, chimera-type galectin 3 (Gal-3) has gained special attention. Gal-3 is upregulated in tumor cells and promotes, as secreted lectin, tumor progression and angiogenesis by receptor clustering [33] and reduction of T-cell functionality [34]. Gal-3 is, therefore, considered as druggable target for anti-cancer therapy [35,36]. Interestingly, Gal-3 is cleaved by matrix-metalloproteinases (MMPs) during tumor progression resulting in a Gal-3 variant, which lacks *N*-terminal amino acid residues 1–62 (Gal-3Δ) [37,38]. Through the partial loss of certain collagen-like domains, the self-association capability is hypothesized to be affected [38,39]. In cancer therapy Gal-3Δ shows synergistic effects with existing anti-tumor drugs and can be used as adjuvant for anti-cancer therapy. Furthermore, Gal-3Δ is suspected to have a higher ligand affinity that may result in shielding carbohydrate ligands and thus acting as negative inhibitor of Gal-3 [40,41,42]. However, multivalent glycan binding specificity studies of Gal-3Δ have not been investigated so far.

We here report, to the best of our knowledge, for the first time the synthesis of poly-LacNAc type 1 oligomers using two recombinant *Leloir*-glycosyltransferases in a one-pot and a sequential synthesis approach. The in-house produced nucleotide sugars UDP-Gal and uridine 5′-diphosphate *N*-acetylglucosamine (UDP-GlcNAc) were provided for an economic synthesis. Selected glycans were further derivatized by a squarate linker for subsequent chemical conjugation to bovine serum albumin. The novel multivalent neo-glycoproteins carrying LacNAc type 1 (Galβ1,3GlcNAc), LacNAc type 2 (Galβ1,4GlcNAc) and hybrid tetrasaccharides were tested for binding of Gal-3 and Gal-3Δ. Interestingly, we gained novel insights into the binding properties and selectivity of Gal-3Δ owing to the neo-glycoproteins, which have been loaded solely with LacNAc type 1 glycans.

## 2. Results and Discussion

### 2.1. Glycan Synthesis

**Scheme 1:**
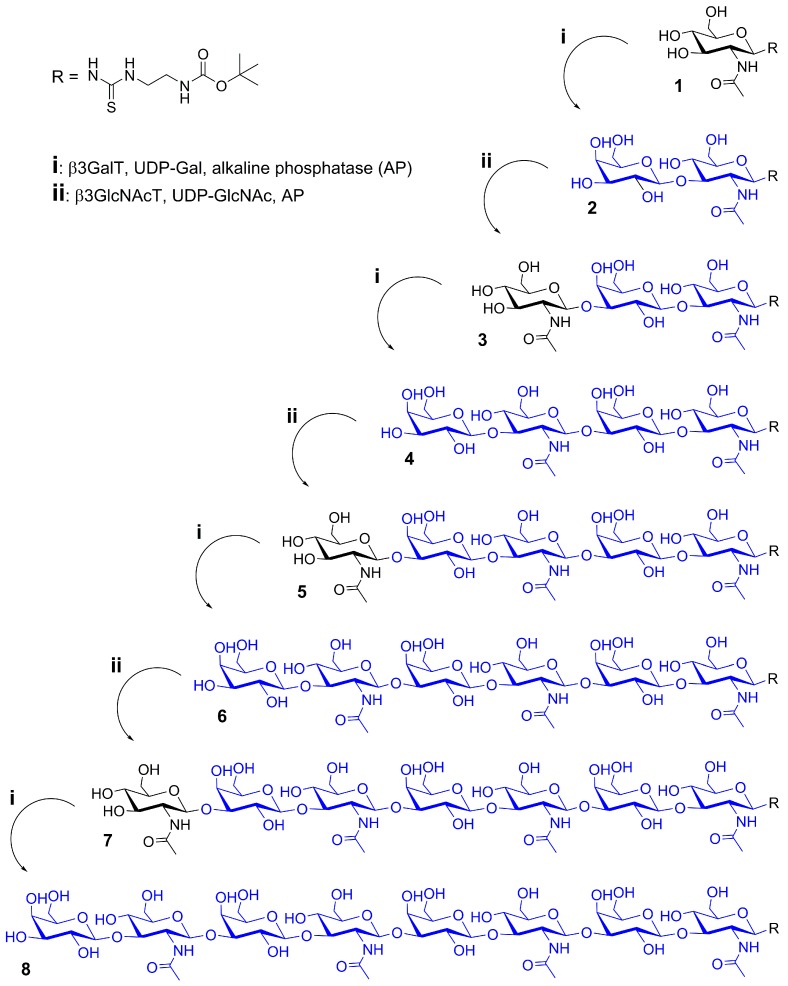
Sequential synthesis of LacNAc type 1 glycan oligomers **2**–**8**.

**Scheme 2:**
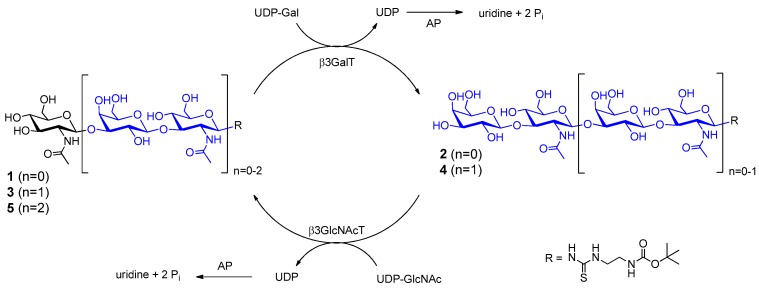
One-pot synthesis of LacNAc type 1 glycan oligomers **2**–**5**.

The aim of the presented work was the synthesis of LacNAc type 1 oligomers by combination of a recombinant β1,3-galactosyltransferase from *E. coli* O55:H7 (β3GalT) and a β1,3-*N*-acetylglucosaminyltransferase from *Helicobacter pylori* (β3GlcNAcT). The bacterial β3GalT [17] was recombinantly expressed in *E. coli* as previously described for other glycosyltransferases [43,44]. The His_6_-tagged enzyme was purified and thoroughly characterized with regard to its pH optimum and requirement for divalent cations (Supporting Information Figure S1). Optimum reaction conditions were found with 100 mM HEPES (pH 7.5) in the presence of 5 mM Mg^2+^ which correspond to those of the bacterial β3GlcNAcT [45,46] previously utilized for one-pot synthesis of LacNAc type 2 oligomers [47]. Accordingly, we combined β3GalT and β3GlcNAcT for LacNAc type 1 oligomer synthesis either in sequential (Scheme 1) or one-pot mode (Scheme 2). The utilized UDP-sugar donors were produced as described previously [18,19]. Both syntheses were started from the chemically derivatized *N*-acetylglucosamine acceptor (GlcNAc-linker-*t*Boc, **1**) together with 1.5-fold (sequential) or two-fold (one-pot) excess of UDP-Gal and UDP-GlcNAc, respectively. Alkaline phosphatase (AP) was added in order to remove UDP as inhibitory byproduct from the reaction.

In the sequential mode (Scheme 1), glycan oligomers up to the pentasaccharide (**2**–**5**) were obtained in high yields with complete acceptor substrate conversion (Table 1). The hexasaccharide (**6**) and octasaccharide (**8**) could only be synthesized in low amounts, despite an additional feed of enzymes (β3GalT, AP) and donor (UDP-Gal). Obviously, β3GalT has a low activity towards higher LacNAc type I oligomeric acceptor substrates (**5**, **7**). In contrast, substrate conversion by β3GlcNAcT could be verified to be completed for each galactose-terminated acceptor (Table 1). The LacNAc type 1 oligomers were isolated by solid-phase extraction. The tetrasaccharide **4** consisting of two LacNAc type 1 repeats was obtained with an overall yield of 95% and utilized for the synthesis of neo-glycoproteins (Section 2.2). The integrity of LacNAc type 1 containing compounds **2**–**8** was confirmed by HPLC-ESI-MS (Table 1, Supporting Information Figure MS2–8). Additionally, we verified the Galβ1,3-linkage of compound **2** by treatment with specific β1,3-galactosidase (BgaC) [48,49] (Supporting Information Figure S12).

**Figure 1:**
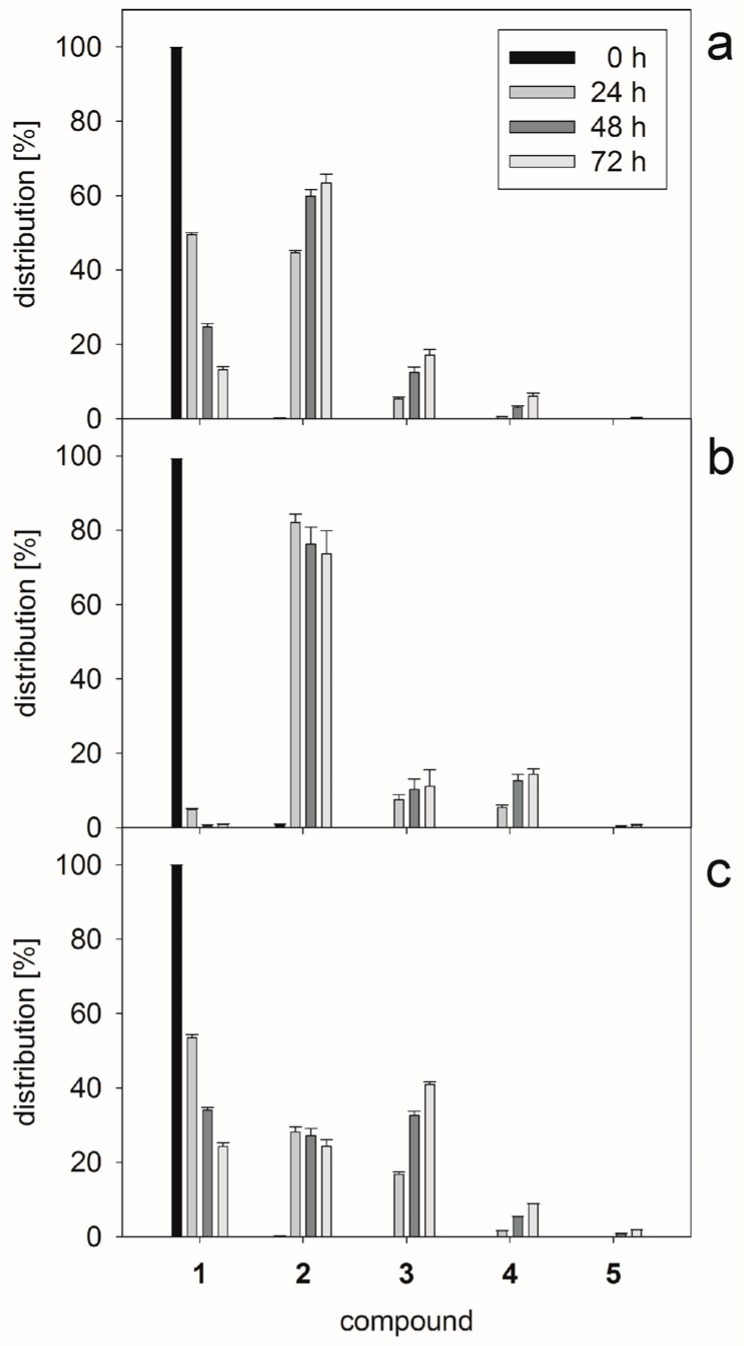
Relative product distribution of LacNAc type 1 glycans **1**–**5** from the respective one-pot syntheses after 24, 48, and 72 h. Different enzyme activity ratios (β3GalT/β3GlcNAcT) of 1:1 (**a**); 5:1 (**b**); and 1:5 (**c**) were adjusted. All reactions contained acceptor **1** (5 mM), while the concentrations for UDP-Gal and UDP-GlcNAc were 10 mM. Samples were analyzed by HPLC and assigned using synthesized standards verified by HPLC-MS.

In our previous investigations, the one-pot combination of β3GlcNAcT and human β1,4-galactosyltransferase (β4GalT) has been successfully established in order to generate LacNAc type 2 glycan oligomers with a high number of LacNAc repeats [47]. In this regard, we investigated one-pot (Scheme 2) reactions at varying enzyme activity ratios of β3GalT and β3GlcNAcT. The reaction mixtures contained acceptor **1** (5 mM), alkaline phosphatase (AP, 10 U) and 10 mM (two-fold excess) of UDP-Gal and UDP-GlcNAc, respectively. The product distribution was analyzed by HPLC after 24, 48, and 72 h reaction time. Different enzyme activity ratios of β3GalT and β3GlcNAcT led to distinct product distributions (Figure 1a–c). Complete conversion of acceptor **1** was reached at an enzyme activity ratio of 5:1 (β3GalT/β3GlcNAcT) (Figure 1b) with the disaccharide **2** as main product and 14% yield for tetrasaccharide **4** after 72 h. Higher amounts of compound **4** were not obtained (Figure 1a,c). However, compound **3** was formed as main product (after 72 h) when β3GlcNAcT was in five-fold excess (Figure 1c). In comparison to the one-pot synthesis using an engineered glycosynthase and β3GlcNAcT [16] the combination of both glycosyltransferases in one-pot synthesis is limited towards the synthesis of the disaccharide and trisaccharide products.

In conclusion, the sequential synthesis approach using β3GalT and β3GlcNAcT results in the effective synthesis of LacNAc type 1 oligomers. In contrast to our previous results with β4GalT and β3GlcNAcT [47] synthesis of higher glycan oligomers was not feasible by one-pot synthesis using β3GalT and β3GlcNAcT.

**Scheme 3:**
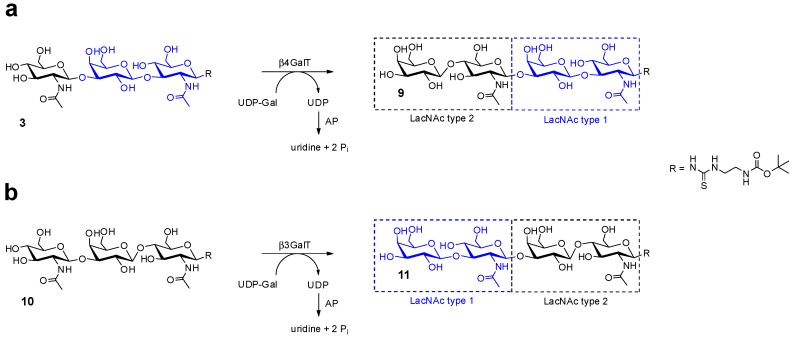
Synthesis of hybrid LacNAc oligomer **9** (**a**) and **11** (**b**).

In addition to tetrasaccharide **4** with a di-LacNAc type 1 structure, we further investigated the synthesis of hybrid oligomers presenting LacNAc type 1 and type 2 motifs at the reducing and non-reducing end of the respective tetrasaccharides (**9** and **11**), depicted in Scheme 3. For this purpose, the trisaccharide **3** was converted by human β4GalT [44,47] to yield compound **9** with terminal LacNAc type 2 (Galβ1,4GlcNAc) motif (Scheme 3). The reaction was completed after 24 h and compound **9** was isolated by solid-phase extraction. Furthermore, trisaccharide **10** was synthesized as previously described [50] and used as an acceptor for β3GalT in order to generate terminal LacNAc type 1 glycosylation motif (tetrasaccharide **11**). The reaction was performed according to the sequential reaction mode and was completed after 72 h. The integrity of hybrid oligomers **9** and **11** was verified by HPLC-ESI-MS (Table 2, Supporting Information Figure MS9 and MS10).

### 2.2. Neo-Glycoprotein Synthesis

Since single carbohydrate-lectin interactions are generally weak, multivalent glycan presentation is essential to trigger multiple glycan-lectin interactions owing to the cluster glycoside effect [51,52,53]. In addition to other platform technologies for multivalent ligand presentation, neo-glycoproteins have been synthesized by chemical coupling strategy using squarate linker chemistry [50,54,55,56]. Multivalent neo-glycoproteins presenting di-LacNAc type 2 tetrasaccharides have been verified as ligands for Gal-3 in previous studies [50]. The herein-synthesized LacNAc type 1-containing tetrasaccharides extend our glycan library and were further used for neo-glycoprotein synthesis.

**Scheme 4:**
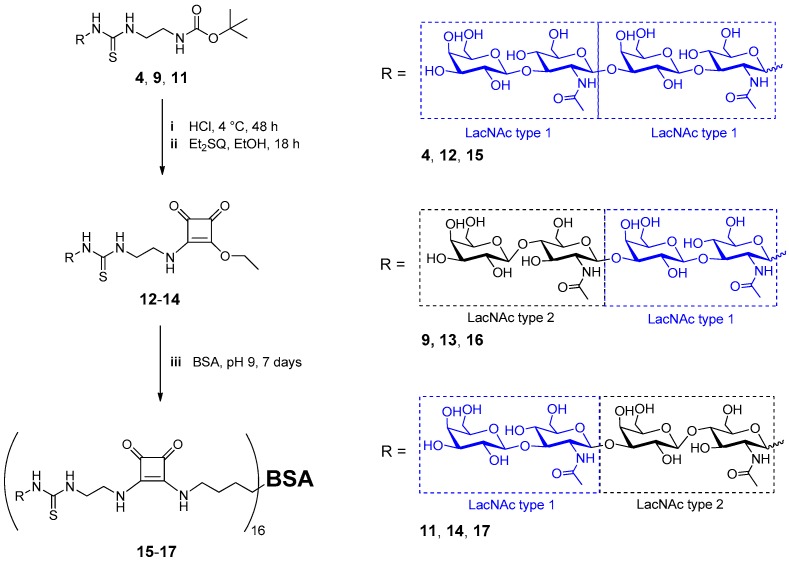
Synthesis of multivalent BSA neo-glycoproteins **15**–**17** by squarate linker chemistry.

In this regard, the tetrasaccharides **4**, **9**, and **11** were chemically conjugated to bovine serum albumin (BSA) to achieve a multivalent ligand presentation on a protein scaffold. Diethyl squarate (3,4-diethoxy-3-cyclobutene-1,2-dione, Et_2_SQ), a homobifunctional linker, was used for the conjugation of the glycans to the amine providing lysine side chains of BSA as described previously [50]. Deprotection of the *t*Boc linker and subsequent amidation of compounds **4**, **9**, and **11** with Et_2_SQ resulted in the corresponding squarate monoamide esters **12**–**14** in high yields (67–88%) after isolation by preparative HPLC (Scheme 4). The purity of isolated compounds **12**–**14** was confirmed by HPLC-ESI-MS (Supporting Information Table S1, Figure MS11–13). In a second step, lysine residues of BSA were reacted with squarate monoamide esters **12**–**14** under slightly alkaline conditions (Scheme 4). The ratio of reactants (compounds **12**–**14** to lysine residues) was adjusted to 0.375. The reaction mixtures were incubated for seven days with gentle shaking at 23 °C.

**Figure 2:**
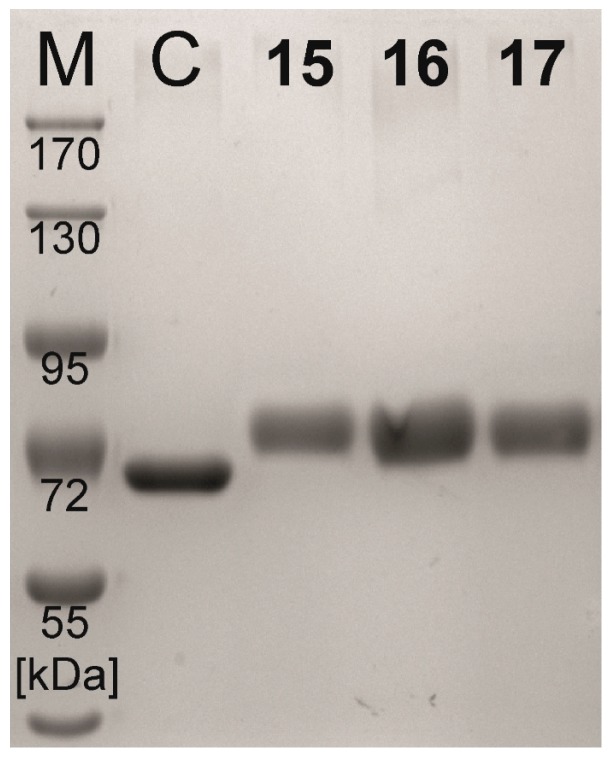
SDS-PAGE analysis of BSA neo-glycoproteins **15**–**17** and non-modified BSA as a control (C); M, PageRuler Prestained Protein Ladder (ThermoFisher Scientific, Darmstadt, Germany).

The resulting BSA neo-glycoproteins **15**–**17** were analyzed by sodium dodecyl sulfate gel electrophoresis (SDS-PAGE) and the 2,4,6-trinitrobenzene sulfonic acid (TNBSA) assay, as described previously [50]. SDS-PAGE analysis (Figure 2) revealed higher molecular masses of the smearing protein bands typically found for glycoproteins. Shifts towards higher molecular masses were 13.2kDa on average. The TNBSA assay determines the number of non-modified lysine residues, from which the number of conjugated glycans can be calculated. BSA (60 mol lysine residues per mol protein) served as a reference. We determined glycan modification densities of 16.6 mol (**15**), 16.5 mol (**16**), and 16.3 mol (**17**) glycans per mol BSA, respectively. The total coupling efficiency, which is the amount of reacted monoamide esters **12**–**14**, ranged between 72% and 74% (Table 3). During the second coupling reaction, ethanol was the leaving group, which corresponds to a loss of 46 g/mol for each attached glycan derivative. Given the molecular mass of squarate monoamide esters **12**–**14** (973 g/mol) and loss of an ethanol during reaction, the molecular mass is expected to be increased by 927 Dalton (Da) per amidated lysine residue. The calculated molecular masses (from TNBSA assay) of neo-glycoproteins **15**–**17** are listed in Table 3 and are in accordance with those from SDS-PAGE.

### 2.3. Galectin Binding Assays

**Figure 3:**
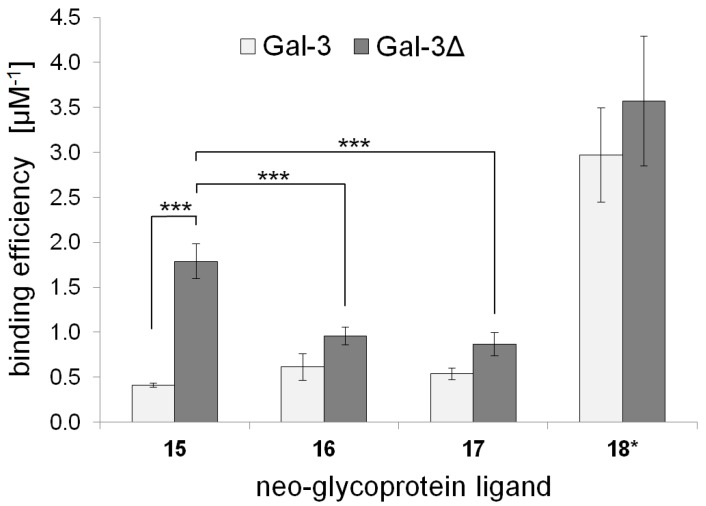
Binding efficiencies of Gal-3 and Gal-3Δ for BSA neo-glycoproteins **15**–**18**. Conjugate **18** featured di-LacNAc type 2 tetrasaccharides and was prepared as previously published [50]. The structure is depicted in the Supporting Information (Figure S16).

Binding properties of human Gal-3 and truncated Gal-3Δ, lacking N-terminal amino acid residues 1–62, were investigated in an enzyme-linked lectin assay (ELLA) using multivalent BSA neo-glycoproteins **15**–**17** as ligands. For this purpose, Gal-3 and Gal-3Δ were expressed in *E. coli* Rosetta (DE3) and purified by affinity chromatography as published previously [50,55,57,58]. The binding of Gal-3 and Gal-3Δ at varying concentrations to immobilized neo-glycoprotein **15**–**17** was quantified by an anti-His_6_ specific antibody. Neo-glycoprotein **18** was utilized as reference [50] due to presented LacNAc type 2 tetrasaccharides (Figure S16, Supporting Information). The corresponding binding curves are depicted in Figure S16 (Supporting Information). For a quantitative comparison, the maximum binding signal (B_max_) and the galectin concentration for half-maximum binding (apparent K_d_ value) were calculated (Table 4). The binding efficiencies (µM^−1^), defined as the ratio of B_max_/K_d_, were determined as direct measure for galectin binding characteristics and are illustrated in Figure 3. The B_max_ values of Gal-3 and Gal-3Δ are quite comparable within the measured standard deviation (Table 4). Both Gal-3 and Gal-3Δ showed the highest B_max_ for neo-glycoprotein **17**. On the contrary, we observed substantial differences of the apparent K_d_ affinity constants, making those the most contributing factors for evaluation of binding efficiencies.

In general, the binding efficiency of Gal-3Δ was consistently increased compared to Gal-3. Furthermore, the binding affinity and efficiency of both Gal-3 and Gal-3Δ was highest when using conjugate **18** [50] as a reference due to a multivalent presentation of LacNAc type 2 glycans as well-known galectin ligands. Among the LacNAc type 1 containing conjugates (**15**–**17**), di-LacNAc type 1 presenting neo-glycoprotein **15** was the preferred ligand of Gal-3Δ (Figure 3). Gal-3Δ binding efficiency for conjugate **15** was more than 4-fold elevated compared to those for Gal-3, being statistically significant with *p* < 0.001 (Figure 3). In contrast, Gal-3Δ binding was significantly (*p* < 0.001) reduced when hybrids of LacNAc type 1 and type 2 are present (neo-glycoproteins **16** and **17**), making them to less favored ligands of Gal-3Δ in our study. Neo-glycoprotein **18** (di-LacNAc type 2) is, however, the most effective ligand for Gal-3 and Gal-3Δ binding. Nevertheless, we demonstrate that neo-glycoprotein **15** is a selective multivalent glycoconjugate for binding of Gal-3Δ with the potential to discriminate between Gal-3 and Gal-3Δ (Table 4, Figure 3). In contrast, the LacNAc type 1/2 hybrid structures (**16**, **17**) and di-LacNAc type 2 glycans (**18**) did not depict significant differences in binding of Gal-3 and Gal-3Δ.

Probably, the carbohydrate recognition domain (CRD) of the Gal-3Δ variant may undergo a conformational change as consequence of its *N*-terminal truncation by MMPs. Our results confirm also previous microarray studies suggesting that LacNAc type 1 glycans are less potent ligands of full-length Gal-3 [59]. LacNAc type 1-presenting neo-glycoprotein **15** may be a promising candidate for the development of anti-cancer vaccines [60,61].

## 3. Materials and Methods

### 3.1. Nucleotide Sugar Synthesis

The nucleotide sugars UDP-Gal and UDP-GlcNAc were provided by enzymatic synthesis, as described previously [19]. A lyophilization step was performed to adjust higher concentrations in starting donor substrate solutions. Concentrations of nucleotide sugars were determined by capillary electrophoresis (Supporting Information Figure S2) [19].

### 3.2. Cloning of β3-Galactosyltransferase Fusion Protein

The synthetic gene encoding β1,3-galactosyltransferase from *Escherichia coli* (*E. coli*) O55:H7 (β3GalT) [17] with C-terminal fusion of lipase pre-propeptide (pp) from *Staphylococcus hyicus* was purchased from GeneArt^TM^ (ThermoFisher Scientific, Darmstadt, Germany). The sequence was codon optimized for recombinant expression in *E. coli*. The functional expression and purification (via His_6_-tag) of the β1,3-galactosyltransferase from *E. coli* O55:H7 failed when cloned into vectors pET-15b and pET-22b, respectively [17]. The lipase pre-propeptide from *Staphylococcus hyicus* [44] was employed as spacer between the enzyme’s coding region and the vector (pET-22b) containing C-terminal His_6_-tag, which enabled immobilized metal-ion affinity chromatography. Digestion of both the expression vector (pET22b) and the synthetic gene using NdeI and XhoI according to manufacturer’s instructions enabled sticky end ligation by usage of T4 DNA ligase (ThermoFisher Scientific, Darmstadt, Germany). Vector pET22b featured a C-terminal His_6_-tag for affinity chromatography, as well as ampicillin marker for selection. Finally, integrity of β3GalTppHis_6_ construct (β3GalT) was confirmed by sequencing (Sequiserve GmbH, Vaterstetten, Germany). The primer sequences were 5′-TAATACGACTCACTATAGG-3′ (forward primer) and 3′-GCTAGTTATTGCTCAGCG-5′ (reverse primer). The pelB leader sequence of pET22b vector was cut by restriction enzyme. Thereby, putative periplasmic secretion of target protein was prevented.

### 3.3. Production of Recombinant Enzymes and Human Galectins

Heterologous protein expression and subsequent purification was carried out as described previously [44,46,47,50,55,62]. The β3GalT fusion protein featuring a C-terminally fused lipase pre-propeptide from *Staphylococcus hyicus* and His_6_-tag (β3GalT) and the β1,3-*N*-acetylglucosaminyltransferases from *Helicobacter pylori* with an *N*-terminal maltose binding protein (β3GlcNAcT) were produced in *E.coli* BL21 (DE3) (Novagen/Merck, Darmstadt, Germany) [45,46,47]. Briefly, *E. coli* BL21 (DE3) cells were grown in 1 L terrific broth (TB) medium (5 L baffled flask, 80 rpm) at 37 °C to an optical density (OD_600_) of 0.6–0.8. After induction with isopropyl-β-D-thiogalactopyranoside (IPTG, 0.1 mM), further cultivation was performed at 20 °C for 22 h. The human β1,4-galactosyltransferase-1 fusion protein (β4GalT) was produced in *E. coli* Shuffle T7 Express (DE3) (NEB, Frankfurt/Main, Germany) as previously published [44,47]. After 24 h cell culture, cells were harvested by centrifugation. A 40% *w*/*v* cell suspension was sonicated, centrifuged (13,000 rpm, 5 min), and the supernatant was used for purification. The His_6_-tagged proteins were isolated using a HisTrap^TM^ HP 5 mL column (GE Healthcare, Munich, Germany) as recommended by the manufacturer. The elution buffer of β3GalT was supplemented with 0.2% (*v*/*v*) Triton^TM^ X-100. MBPTrap^TM^ HP 5 mL column (GE Healthcare) was applied for purification of MBP-tagged protein (β3GlcNAcT). The buffer of eluted β3GalT was exchanged by dialysis against phosphate buffer (100 mM NaH_2_PO_4_, 500 mM NaCl, 5 mM DTT, pH 7.5).

The human galectin fusion constructs His_6_-Gal-3 (Gal-3) and the truncated version His_6_-Gal-3 (Gal-3∆1-62, Gal-3∆) were produced in *E. coli* Rosetta (DE3) pLysS (Novagen/Merck, Darmstadt, Germany) and purified, as described previously [50,55,57]. Immobilized metal-ion affinity chromatography (IMAC) was carried out via HisTrap^TM^ HP 5 mL columns as mentioned above. The buffer of eluted galectins was exchanged by dialysis against phosphate buffered saline containing ethylenediaminetetraacetic acid (2 mM, EPBS, pH 7.5). Protein concentrations were determined by a Bradford assay (Roti^®^-Quant, Carl Roth, Karlsruhe, Germany) using bovine serum albumin for calibration.

### 3.4. Enzyme Activity Assays

The β3GalT activity was determined in different buffer systems (MES, MOPS, HEPES, Tris and glycine, 100 mM each, pH 5.5–10.0) containing 25 mM KCl, 5 mM of divalent cations (Mg^2+^, Mn^2+^, Zn^2+^, Cu^2+^, Ca^2+^, Co^2+^; applied as chloride salts), 5 mM acceptor **1**, 6.5 mM UDP-Gal, purified β3GalT (27 µg), and 3 U alkaline phosphatase (Fast AP, ThermoFisher, Darmstadt, Germany). Additionally, activity was proven under conditions for preparative syntheses using 100 mM HEPES (pH 7.6) containing 25 mM KCl, 20 mM MgCl_2_, 10 mM UDP-Gal, 10 mM UDP-GlcNAc, and 5 mM acceptor **1**, 10 U alkaline phosphatase (AP, from ThermoFisher), and purified β3GalT (27 µg). The β3GlcNAcT activity was assayed under the same conditions as described for the β3GalT with the exception that 5 mM of compound **2** was used as substrate for β3GlcNAcT (2.5 µg). The β4GalT activity was determined as previously described [44,47] in 100 mM HEPES-NaOH (pH 7.5) containing 25 mM KCl, 6.5 mM MnCl_2_, 6.5 mM UDP-Gal, and acceptor **1**. The total volume of all reactions was adjusted to a final volume of 50 µL that was incubated at 30 °C. The reactions were stopped after 0, 5, 10, 15, 30, 60, and 120 min by short heating (95 °C for 5 min) followed by a centrifugation step (5 min, 13,000 rpm) to remove denatured protein. The supernatant was diluted 1:5 in water and analyzed by HPLC. The retention time of the compounds was 26.67 min (**1**, acceptor) and 21.70 min (**2**, product), respectively, as shown in Figure S3 (Supporting Information). Volumetric activity (U/mL) was deduced from the linear slope area. Specific enzyme activity (U/mg) was calculated under consideration of protein concentration (mg/mL). One unit (1 U) was defined as the amount of enzyme that converts one µmol substrate per minute.

### 3.5. One-Pot Synthesis of Poly-LacNAc Type 1 Oligomers

Compound **1** (GlcNAc-linker-*t*Boc) was synthesized as described previously [46] and used as starting material for glycan synthesis. The one-pot syntheses were carried out on an analytical scale in 100 mM HEPES-NaOH (pH 7.5) supplemented with 25 mM KCl and 5 mM acceptor **1**. The reactions contained a two-fold excess of UDP-Gal and UDP-GlcNAc (both 10 mM), 20 mM MgCl_2_, 1 mM DTT, and AP (10 U). Varying enzyme activity ratios (β3GalT/β3GlcNAcT) of 1:5, 1:1, and 5:1 were adjusted, ranging between 2 and 10 mU of appropriate enzymes.

### 3.6. Sequential Synthesis of Poly-LacNAc Type 1 Oligomers

The sequential synthesis was started with acceptor **1** (4.3 mg, 10.1 µmol), which was consecutively treated alternately with purified β3GalT or β3GlcNAcT to create a growing oligosaccharide chain of repeating LacNAc type 1 units. The consecutive batches (2.02 mL) contained 5 mM acceptor substrate, 100 mM HEPES-NaOH (pH 7.5), 25 mM KCl, 20 mM MgCl_2_, 10 mM UDP-Gal or 10 mM UDP-GlcNAc, AP (10 U), and 150 mU/mL purified enzyme (β3GalT or β3GlcNAcT). After 18 h (compounds **2**–**5**, **7**) or 48 h (**6**, **8**), reactions were stopped by heat and centrifugation as described above. HPLC analysis confirmed the presence of elongated glycans as previously described [47,55]. Removal of denatured enzyme was achieved by ultrafiltration (VivaSpin^®^ 20, MWCO 30 kDa, Sartorius Stedim Biotech, Goettingen, Germany). Buffer components and UDP sugars were removed by solid-phase extraction using Sep-Pak^®^ C18 3cc Vac Cartridges (Waters Corporation, Eschborn, Germany) in order to obtain pure products. Compound **3** was further treated with β4GalT as described previously [50] to generate LacNAc type 2 terminated tetrasaccharide **9**, which was isolated by solid-phase extraction as described above. Compound **11** was obtained by treating compound **10** [50] with β3GalT under given reaction conditions in order to add a terminal LacNAc type 1 motif as described above.

### 3.7. HPLC Analysis and Mass Spectrometry

Activity assays and synthetic reactions were analyzed on a Dionex system using a column packed with MultoKrom 100-5 C18 resin (250 mm × 4 mm, CS-Chromatographie, Langerwehe, Germany). As eluent 15% (*v*/*v*) acetonitrile dissolved in MilliQ water was used at a flow rate of 1 mL∙min^−1^. LacNAc oligomers could be detected due to presence of the UV-active linker at 254 nm. Mass analysis of oligomers **2**–**11** was performed by electrospray ionization mass spectrometry (ESI-MS). Pure compounds (0.1–0.2 mM), partly supplemented with 0.5 µL ammonium hydroxide, were analyzed on a Multospher 120 RP 18 HP-3μ HPLC column (60 mm × 2 mm, CS-Chromatographie) at a flow rate of 0.2 mL min^−1^ using MS-grade acetonitrile/water (50:50) as mobile phase. Mass data were collected with a Finnigan Surveyor MSQ Plus (Thermo Scientific, needle voltage, 4 kV; temperature, 400 °C; cone voltage, 100 V; negative mode). Mass/charge-ratio (*m*/*z* ratio) was the fundamental unit for detection of target molecules.

### 3.8. Neo-Glycoprotein Synthesis

Oligomer functionalization with 3,4-diethoxy-3-cyclobutene-1.2-dione (diethyl squarate, Et_2_SQ) was performed as reported previously [50]. In short, deprotection of compounds **4** (2.20 µmol, 2.09 mg), **9** (1.99 µmol, 1.89 mg) and **11** (2.55 µmol, 2.42 mg) was performed in 1 M HCl for 48 h at 4 °C. After neutralization (Dowex^®^ 66 free base, Sigma Aldrich, Steinheim, Germany), a four-fold excess of both the diethyl squarate (Et_2_SQ, 7.96–10.20 µmol, 1.35–1.73 mg) and triethylamine (Et_3_N, 7.96–10.20 µmol, 0.81–1.03 mg) was adjusted with regard to deprotected oligosaccharides. Reactions were performed in 50% aqueous ethanol buffered at pH 7.0 (35 mM HEPES) and analyzed by analytical HPLC as described before [50]. Semi-preparative HPLC (Supporting Information Figure S13–S15) was performed for isolation of pure squarate monoamide esters **12**–**14** followed by mass spectrometry analysis (Supporting Information Figures MS 11–13). Squarate monoamide esters **12**–**14** (0.39 µmol) were mixed with delipidated BSA (1.16 mg, 1.04 µmol lysine residues) in the second coupling reaction using borate buffer (50 mM Na_2_B_4_O_7_, pH 9.0). After seven days, the number of modified lysine residues of neo-glycoproteins **15**–**17** was quantified by TNBSA assay as described before [50]. After filtration and buffer exchange (deionized water, VivaSpin^®^ 500, Sartorius Stedim Biotech, Goettingen, Germany), neo-glycoproteins **15**–**17** were further analyzed by SDS-PAGE [50].

### 3.9. Galectin Binding Assays and Statistical Analysis

Neo-glycoproteins **15**–**17** were applied as ligands for Gal-3 and Gal-3Δ in an ELLA-type assay. Appropriate amounts of **15**–**17** (0.1 µM in PBS, 50 µL, 5 pmol per well) were immobilized overnight in F16 Maxisorp NUNC-Immuno Modules (Thermo Scientific, Roskilde, Denmark). After three washing steps using PBS supplemented with 0.05% (*v*/*v*) Tween 20 (PBST), a solution containing 2% (*w*/*v*) delipidated BSA dissolved in PBS was used to block residual binding sites. A subsequent washing step was performed before Gal-3 and Gal-3Δ were added at different concentrations (1–5000 nM, 50 µL) and incubated for 1 h at room temperature. Removal of non-bound galectin was achieved by three-fold PBST washing. Peroxidase conjugated anti-His_6_-IgG2a from mouse (Roche Diagnostics, Mannheim, Germany) was diluted in PBS (1:4000) and added to each well (50 µL, 1 h, room temperature). After three additional washing steps (PBST) reaction of IgG-conjugated peroxidase was initiated by 3,3′5,5′-tetramethylbenzidine (TMB) One (Kem-En-Tec, Taastrup, Denmark) substrate solution (50 µL) and stopped by 3 M HCl (50 µL). Galectin binding signal was quantified by measuring optical density at 450 nm using Spectra Max Plus (Molecular Devices, Biberach, Germany) plate reader. SigmaPlot software was utilized for further analysis of half-maximal binding (apparent *K_d_*) and maximum binding signal (*B*_max_), outlined in Equations (1) and (2). Data was statistically analyzed using a *t*-test with a confidence interval with *p* < 0.001 (*n* = 3).

(1) $$Y=\frac{B_{\max}\cdot X}{k_{d}+X}$$

The determination of half-maximal (*K_d_*) and maximal binding (*B*_max_) signals were calculated by using SigmaPlot software (SigmaPlot 10, Systat Software GmbH, Erkrath, Germany); *Y*, binding signal; *X*, galectin concentration (µM).

(2) $$\begin{array}{l} binding\;efficiency=\frac{B_{\max}}{k_{d}}\;(A)\\ \sigma_{binding\;efficiency\;}=(\frac{\sigma_{B_{\max}}}{B_{\max}}+\frac{\sigma_{k_{d}}}{k_{d}})\cdot binding\;efficiency\;(B) \end{array}$$

where (*A*) is the determination of binding efficiency and the corresponding standard deviation (*σ*, *B*) is the quotient of the maximal binding signal (*B*_max_) and the half-maximal binding signal.

## 4. Conclusions

We present here, for the first time, an efficient synthesis of LacNAc type 1 oligomers by the sequential use of two recombinant *Leloir*-glycosyltransferases. Tetrasaccharide glycans containing either di-LacNAc type 1 or LacNAc type 1/type 2 hybrid structures were utilized for the synthesis of multivalent BSA neo-glycoproteins. Galectin binding assays reveal the di-LacNAc type 1 glycan motif as highly selective for binding of Gal-3∆, the cancer related *N*-terminally truncated version of full-length Gal-3. LacNAc type 1 presenting neo-glycoproteins may, thus, be useful candidates for tracing Gal-3∆ during tumor progression.

## Figures and Tables

**Table 1:** Sequential synthesis of glycan oligomers **2**–**8** and their mass spectrometry analysis.

Compound	Amount (µmol)	Stepwise Enzymatic Conversion (%)	Stepwise Molar Yield (%)	Calculated *m*/*z*	Observed *m*/*z*
**2**	9.97	100.0	98.3	583.2 ^a^	583.1 ^a^
**3**	9.82	100.0	98.5	786.3 ^a^	786.1 ^a^
**4**	9.61	100.0	97.9	948.4 ^a^	948.2 ^a^
**5**	9.10	100.0	94.7	575.3 ^b^	575.3 ^b^
**6**	2.29	25.2	24.2	656.3 ^b^	656.5 ^b^
**7**	2.22	100.0	96.8	757.8 ^b^	758.0 ^b^
**8**	0.43	20.9	19.5	838.8 ^b^	839.1 ^b^

^a^ [M − H]^−^; ^b^ [M − 2H]^2−^

**Table 2:** Synthesis and MS analysis of LacNAc type 1/2 hybrid oligomers **9** and **11**.

Compound	Amount (µmol)	Stepwise Enzymatic Conversion (%)	Stepwise Molar Yield (%)	Calculated *m*/*z*	Observed *m*/*z*
**9**	4.0	100	95.9	948.4 ^a^	948.3 ^a^
**11**	4.0	100	97.5	948.4 ^a^	948.5 ^a^

^a^ [M − H]^−^

**Table 3:** Synthesis and analysis of BSA neo-glycoproteins **15**–**17**.

Compound	Coupling Efficiency (%)	Conjugated Glycans (TNBSA Assay) (mol/mol BSA)	Molecular Mass (Calculated) (kDa)	Molecular Mass (SDS-PAGE) (kDa)
**BSA**	-	0.0 ± 0.21	66.4	65.4
**15**	73.7	16.6 ± 0.20	81.8	78.8
**16**	73.3	16.3 ± 0.10	81.5	78.3
**17**	72.4	16.5 ± 0.26	81.7	78.6

**Table 4:** Binding behavior of Gal-3 and Gal-3Δ to BSA neo-glycoproteins **15**–**18**.

Neo-Glycoprotein	Galectin Binding Efficiency (µM^−1^)		Maximal Binding Signal (B_max_)		Apparent K_d_ Value (µM)	
	Gal-3	Gal-3Δ	Gal-3	Gal-3Δ	Gal-3	Gal-3Δ
**15**	0.41 ± 0.02	1.79 ± 0.20	1.05 ± 0.02	1.00 ± 0.04	2.56 ± 0.09	0.56 ± 0.04
**16**	0.61 ± 0.07	0.96 ± 0.13	1.11 ± 0.10	1.08 ± 0.03	1.81 ± 0.36	1.13 ± 0.12
**17**	0.54 ± 0.15	0.87 ± 0.10	1.15 ± 0.10	1.10 ± 0.04	2.14 ± 0.41	1.27 ± 0.10
18 *	2.97 ± 0.52	3.57 ± 0.72	1.01 ± 0.09	1.00 ± 0.06	0.34 ± 0.09	0.28 ± 0.04

* neo-glycoprotein **18** was prepared as previously published [50]. The structure is depicted in the (Supporting Information Figure S16).

## Supplementary Materials

The following are available online: **Figure S1**. Characterization of novel β3GalT construct; **Figure S2**. Quantification of nucleotide sugars; **Figures S3–S11**. HPLC chromatograms for the synthesis of compounds **2**–**10**; **Figure S12**. Specific β1,3-galactosidase digestion of LacNAc type 1 structure; **Figures S13–S15**. Isolation of squarate monoamide esters **11**–**13**; **Table S1**. Mass spectrometry analysis of compounds **11**–**13**; b Graphs for galectin binding to neo-glycoproteins **15**–**17**; **Figures MS1–MS13**. Mass spectrometry spectra for all synthesized compounds.
