# Supplementary material for: Enzymatic Synthesis of N-Acetyllactosamine (LacNAc) Type 1 Oligomers and Characterization as Multivalent Galectin Ligands
Source: Molecules. 2017 Aug 10;22(8):1320. doi: 10.3390/molecules22081320 (PMC6152129; doi:10.3390/molecules22081320)
Supplement: Supplementary file 1 [file molecules-22-01320-s001.pdf]

molecules
MDPI

Supporting Information

Enzymatic synthesis of *N*-acetyllactosamine (LacNAc) type 1 oligomers and characterization as multivalent galectin ligands

Thomas Fischöder [1,†], Dominic Laaf [1,†], Carina Dey [1] and Lothar Elling [1,*]

[1] Laboratory for Biomaterials, Institute for Biotechnology and Helmholtz-Institute for Biomedical Engineering, RWTH Aachen University, Pauwelsstraße 20, 52074 Aachen, Germany; t.fischoeder@biotec.rwth-aachen.de, d.laaf@biotec.rwth-aachen.de, c.dey@biotec.rwth-aachen.de

† These authors contributed equally to this work
* Correspondence: l.elling@biotec.rwth-aachen.de; Tel.: +49-241-80-28350

Academic Editor: name
Received: date; Accepted: date; Published: date

Abstract: Repeats of the disaccharide unit *N*-acetyllactosamine (LacNAc) occur as type 1 (Galβ1,3GlcNAc) and type 2 (Galβ1,4GlcNAc) glycosylation motifs on glycoproteins and glycolipids. The LacNAc motif acts as binding ligand for lectins and is involved in many biological recognition events. To the best of our knowledge, we present for the first time the synthesis of LacNAc type 1 oligomers using recombinant β1,3-galactosyltransferase from *Escherichia coli* and β1,3-*N*-acetylglucosaminyltranferase from *Helicobacter pylori*. Selected tetrasaccharide glycans presenting LacNAc type 1 repeats or LacNAc type 1 at the reducing or non-reducing end, respectively, were conjugated to bovine serum albumin as protein scaffold by squarate linker chemistry. The resulting multivalent LacNAc type 1 presenting neo-glycoproteins were further studied for specific binding of the tumor associated human galectin 3 (Gal-3) and its truncated counterpart Gal-3Δ in an enzyme-linked lectin assay (ELLA). We observed a significantly increased affinity of Gal-3Δ towards the multivalent neo-glycoprotein presenting LacNAc type 1 repeating units. This is the first evidence for differences in glycan selectivity of Gal-3Δ and Gal-3 and may be further utilized for tracing Gal-3Δ during tumor progression and therapy.

Keywords: neo-glycoproteins; biocatalysis; LacNAc type 1; chemo-enzymatic synthesis; one-pot; sequential; glycosyltransferase; galectin-3; multivalency.

Enzyme characterization

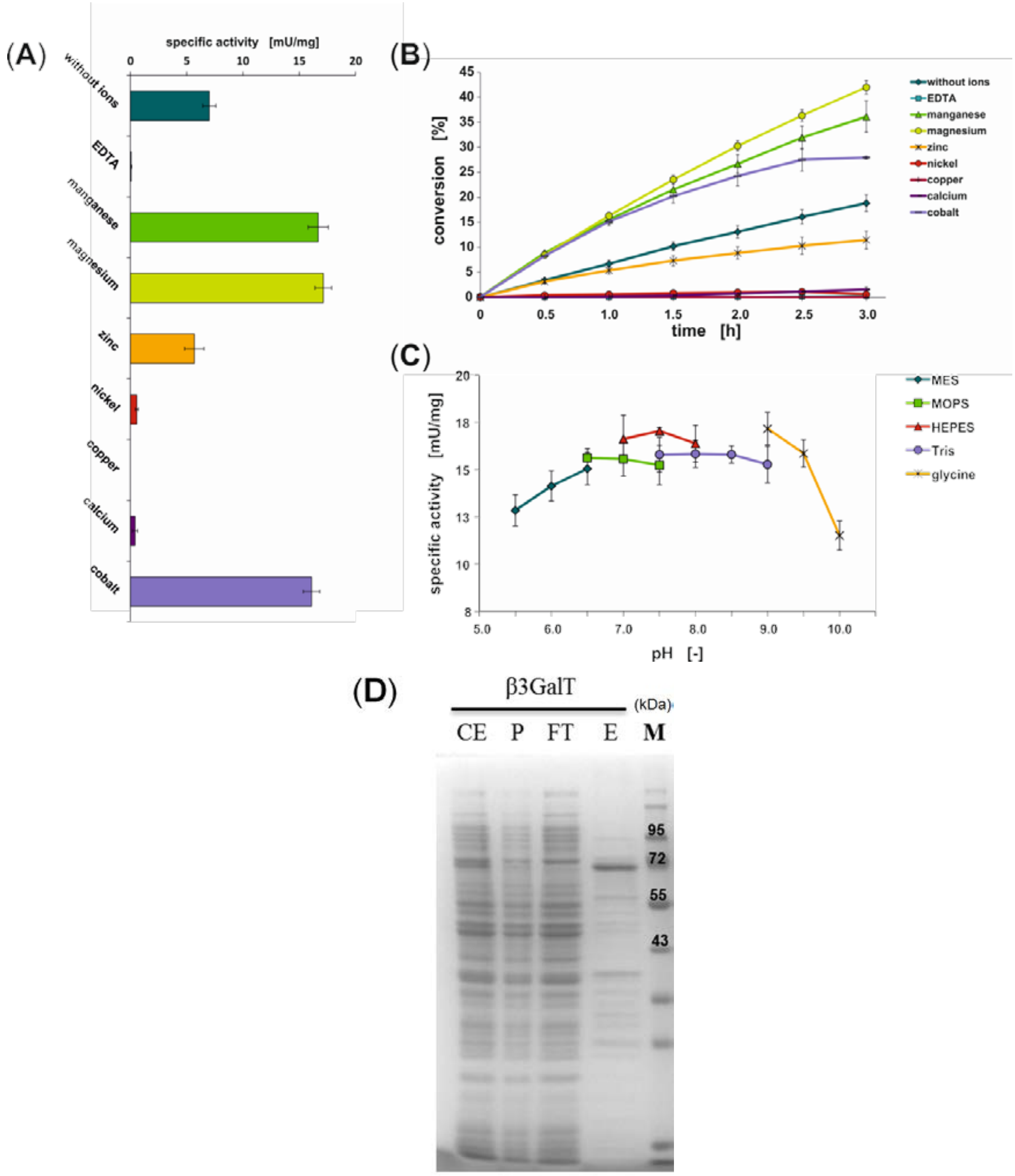

Figure S1. Characterization of β3GalT from *E.coli* O55:H7 with regard to divalent cations (**A**, **B**) and pH (**C**). **A** and **B**: Reactions (100 mM HEPES [pH 7.5], 25 mM KCl, 6.5 mM UDP-Gal, 5 mM acceptor **1**, 3 U alkaline phosphatase, 20 µg β3GalT and 5 mM of indicated divalent cations Mn^{2+}, Mg^{2+}, Zn^{2+}, Ni^{2+}, Cu^{2+}, Ca^{2+} and Co^{2+}) were incubated at 30 °C up to 3 h and stopped by heat at certain time points (95 °C, 5 min) followed by centrifugation (13,400 rpm, 10 min) to remove denatured enzymes. Product formation was monitored by RP-HPLC. **C**: Reactions (6.5 mM UDP-Gal, 5 mM $MgCl_2$, 5 mM acceptor **1**, 3 U alkaline phosphatase, 20 µg β3GalT and 100 mM of indicated buffers MES, MOPS, HEPES, Tris and glycine at given pH) were incubated at 30 °C up to 140 min. Reaction stop and analysis was performed as described above. With regard to the specific activity calculation, one unit [1 U] was defined as the amount of enzyme that converts one µmol substrate per minute. **D**: Reducing gel electrophoresis showing the separated protein samples of crude extract (CE), pellet (P), flow-through (FT) and eluate (E) of an affinity chromatography in order to isolate β3GalT. We found no inclusion bodies or lost protein in the P and FT fractions. Technically pure β3GalT was the dominant band in the eluate (~70 kDa).

The yield of isolated protein per liter culture medium was 52.5 mg for β3GalT. With glycoside acceptor **1**, the specific enzymatic activity was 30.8 mU/mg. The total amount of enzyme was 1.62 U.

Nucleotide sugar quantification

Nucleotide sugar concentration was determined using capillary electrophoresis method as previously described by Wahl *et al.* [1] using a capillary system from Agilent (CE7100) and OpenLAB CDS ChemStation software from Agilent (Rev. C01.07 [27]). Changes were made as follows: Separation of analytes was performed on a fused-silica capillary ID 50 with an effective length of 56 cm and a total length of 64.5 cm. The capillary cassette was tempered to 25 °C. Post conditioning of the capillary was performed by a flushing step with 0.1 M NaOH followed by distilled H_2O for each 60 s and an equilibration step with the electrophoresis buffer (50 mM ammonium acetate pH 9.2 (NaOH) with 1 mM EDTA) for 120 s with approximately 950 mbar (14 psi). Samples were cooled to 4°C by an external closed cycle cooling system and hydrodynamically injected by 35 mbar (0.5 psi) for 5 s. The separation voltage was adjusted to 30 kV in order to maintain an appropriate separation in a suitable time (20 min).

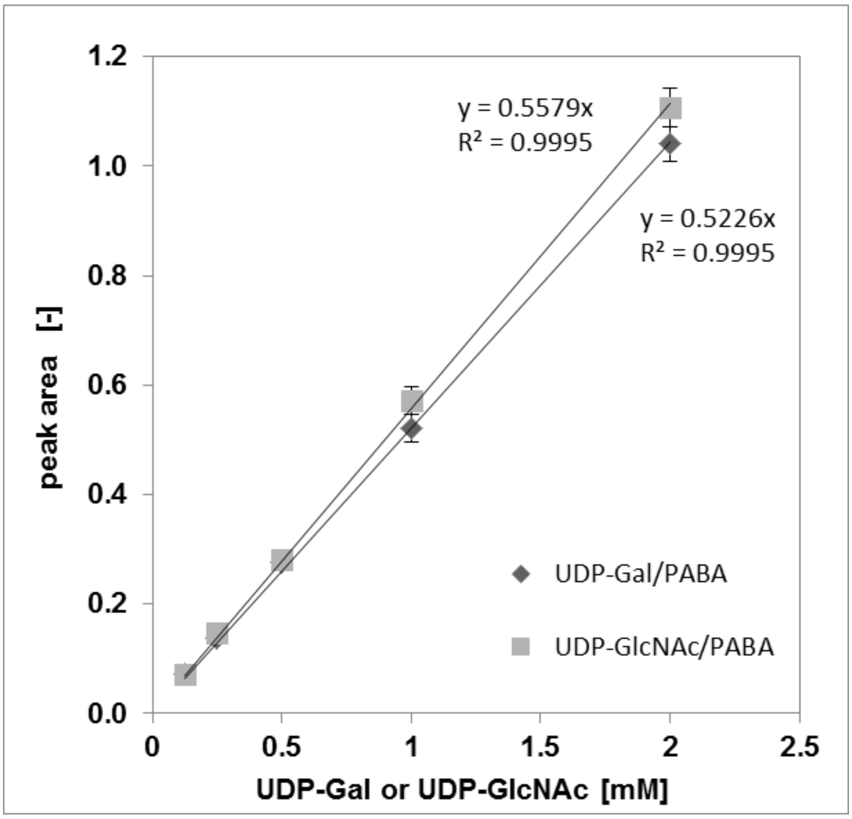

Figure S2. Calibration of UDP-Gal and UDP-GlcNAc concentration by relative peak area. Peak areas were set in relation to the peak area of the internal standard para-amino benzoic acid (PABA, 1 mM, Sigma-Aldrich, Deisenhofen, Germany).

Glycan synthesis

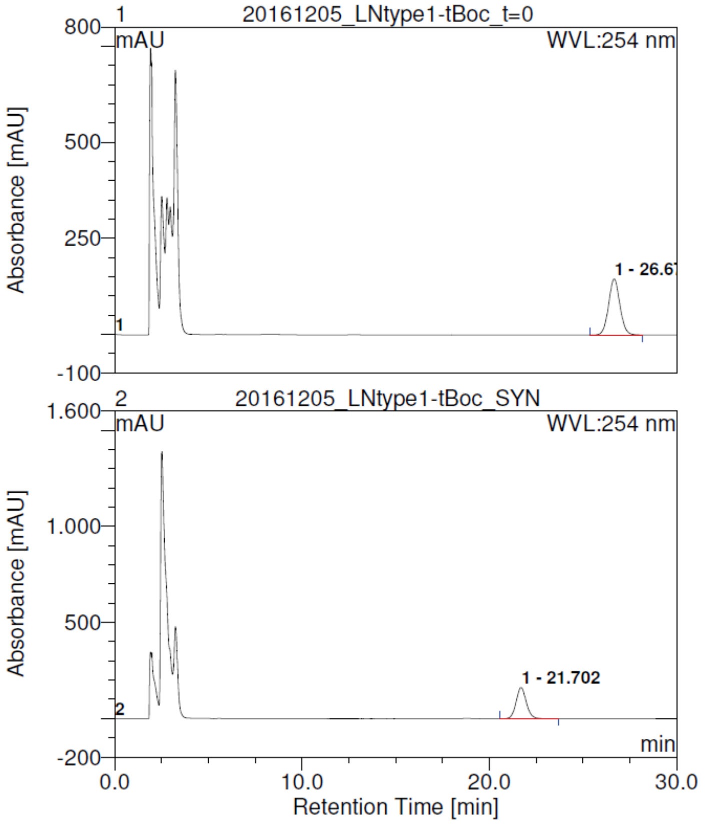

Figure S3. Synthesis of compound **2** (retention time: **1** (t_R=26.67 min), **2** (t_R=21.70 min)).

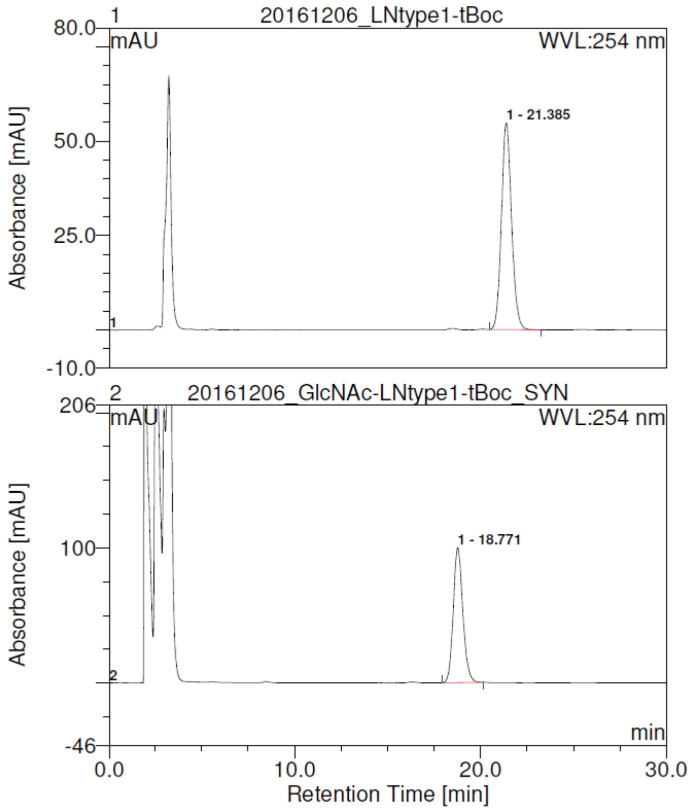

Figure S4. Synthesis of compound **3a** (retention time: **2** (t_R=21.37 min), **3** (t_R=18.77 min).

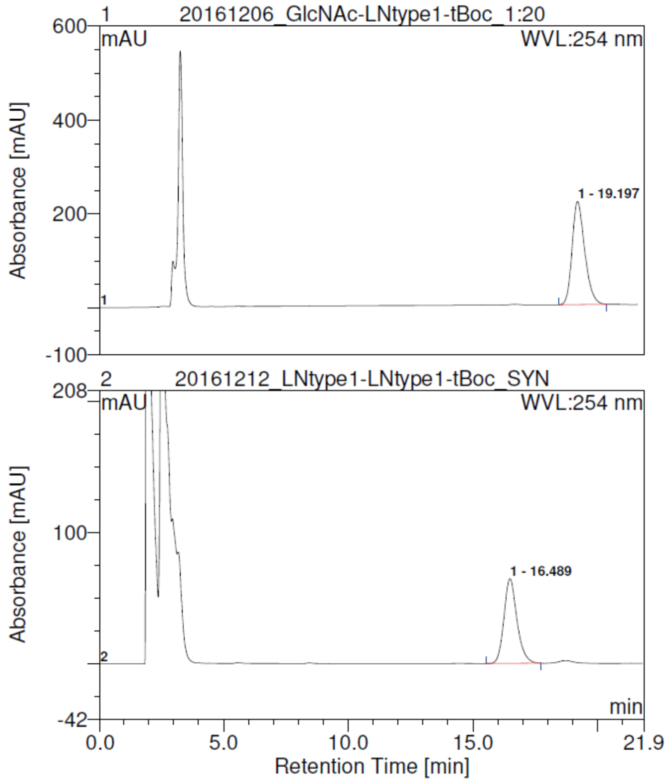

Figure S5. Synthesis of compound **4** (retention times: **3** (t_R=19.20 min), **4** (t_R=16.49 min)).

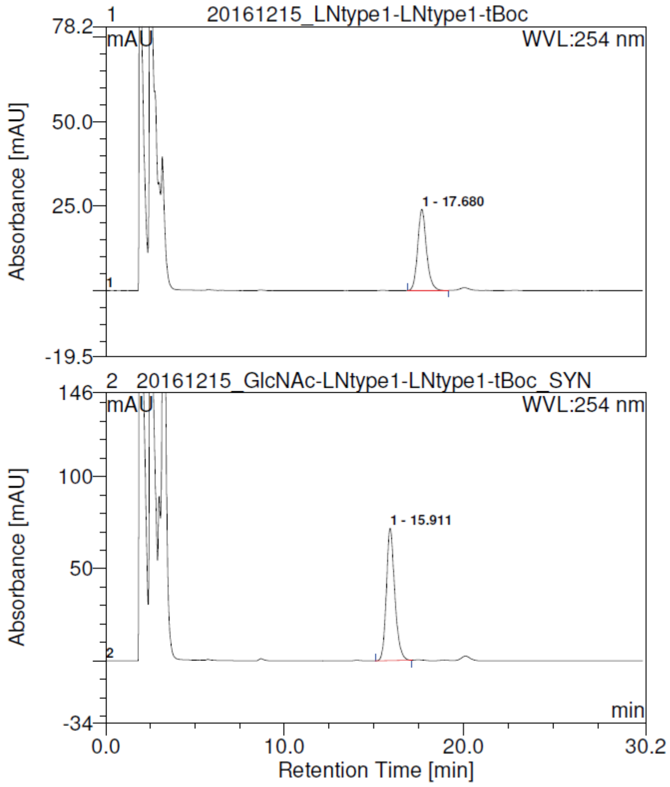

Figure S6. Synthesis of compound **5** (retention times: **4** (t_R=17.68 min), **5** (t_R=15.91 min)).

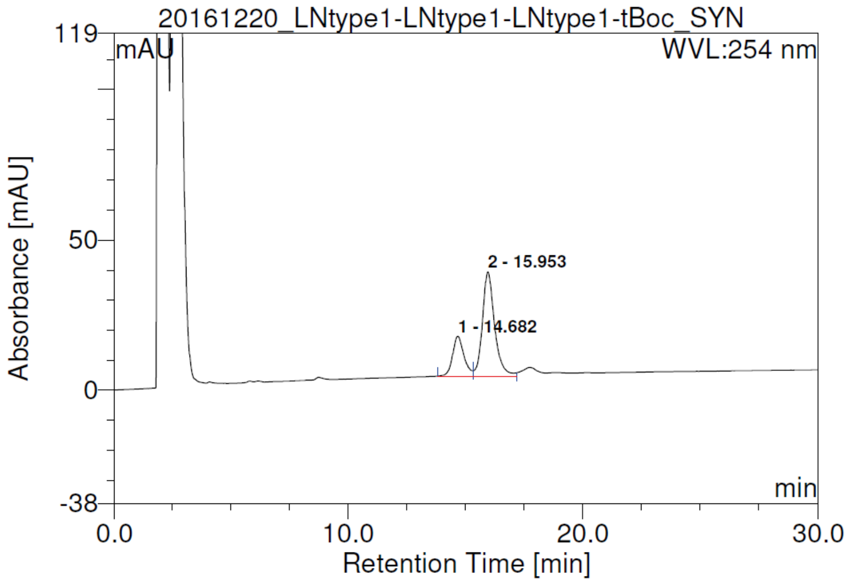

Figure S7. Synthesis of compound **6 (**retention times: **5** (t_R=15.95 min), **6** (t_R=14.68 min)).

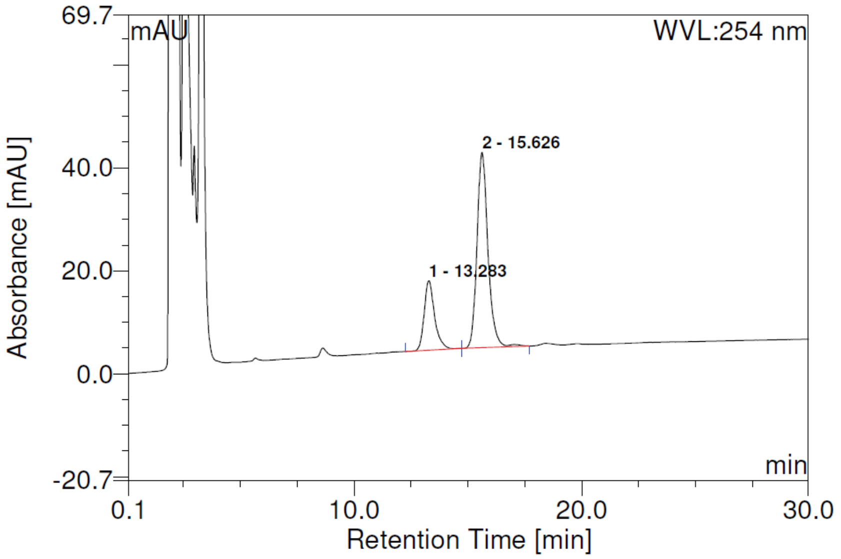

Figure S8. Synthesis of compound **7** (retention times: **5** (t_R=15.63 min), **7** (t_R=13.28 min)).

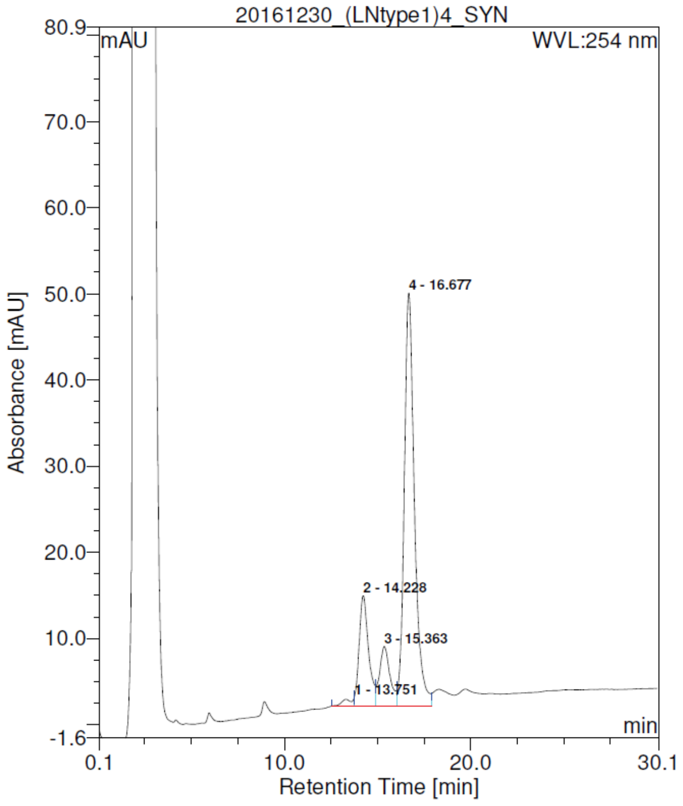

Figure S9. Synthesis of compound **8** (retention times: **5** (t_R=16.68 min), **6** (t_R=15.36 min), **7** (t_R=14.23 min), **8** (t_R=13.75 min)).

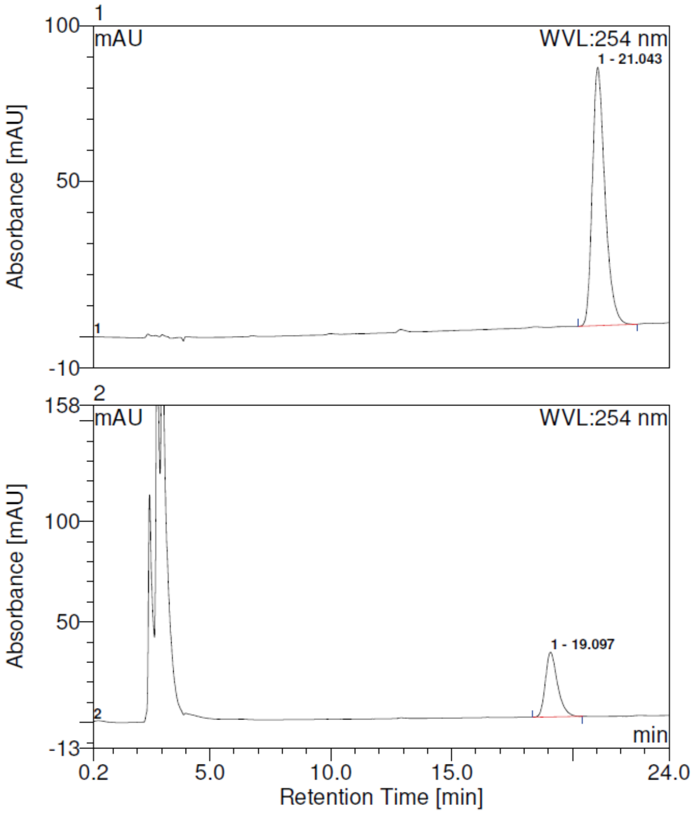

Figure S10. Synthesis of compound **9** (retention times: **3** (t_R=21.04 min, **9** (t_R=19.10 min)).

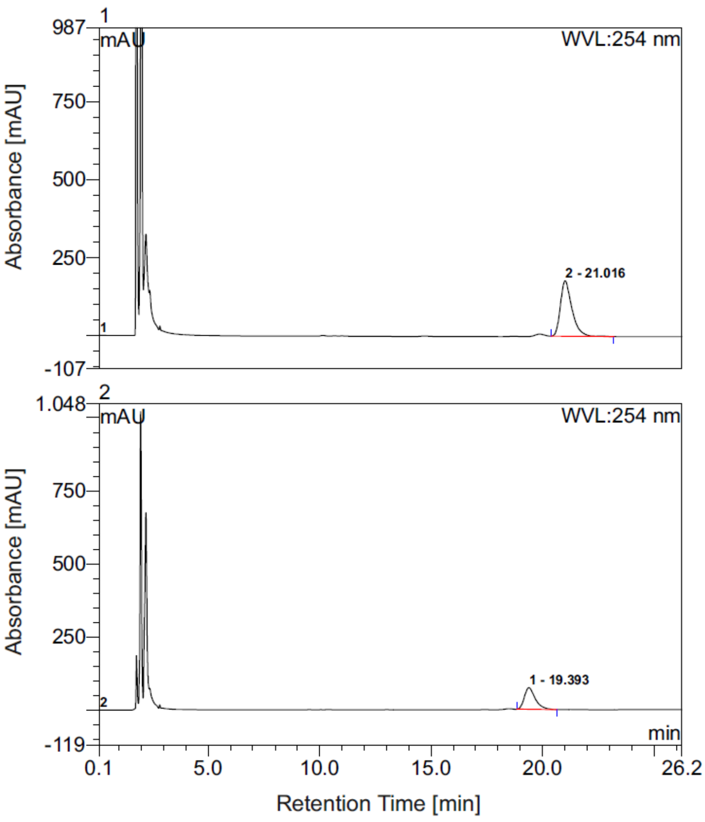

Figure S11. Synthesis of compound **11** (retention times: **10** (t_R=21.02 min, **11** (t_R=19.39 min)).

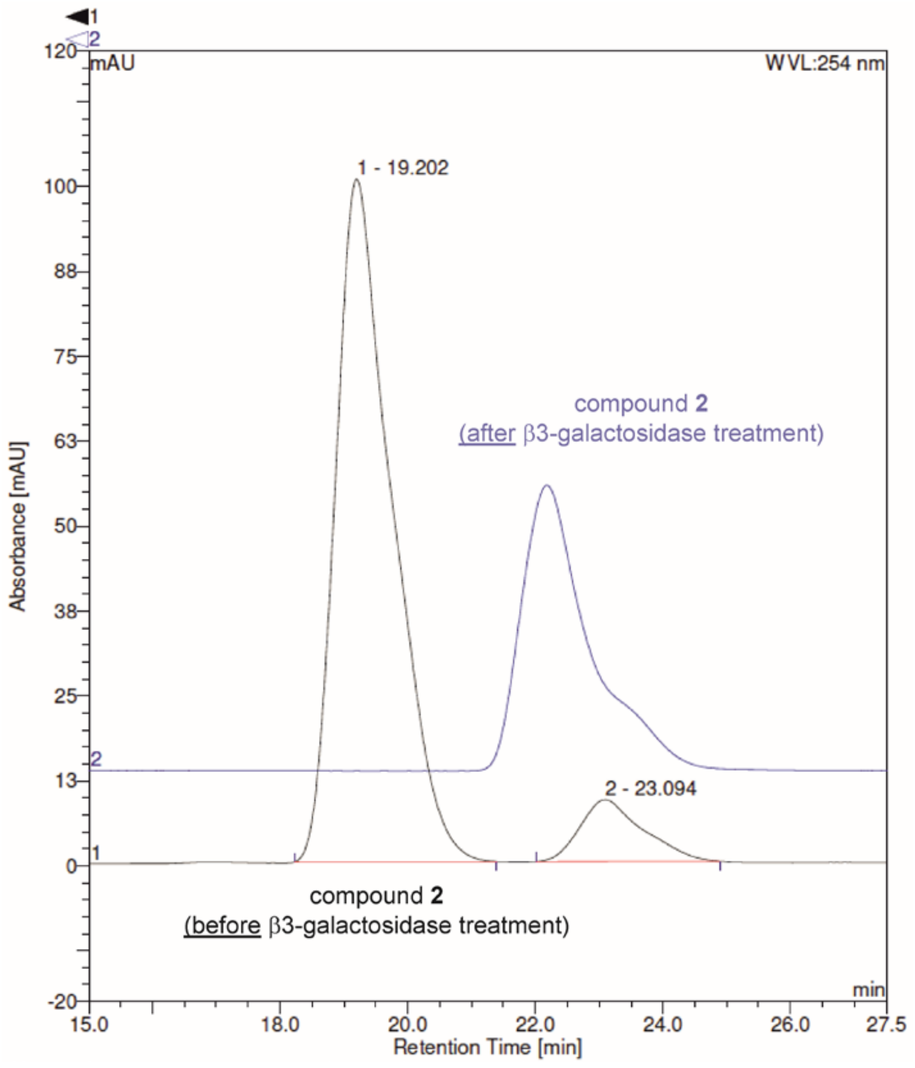

Figure S12. Treatment of compound **2** (t_R=19.20 min) with specific β3-galactosidase (BgaC) [2,3] in order to verify Galβ1,3-linkage. The reaction was incubated for 24 h at 30 °C (50 mM citrate-Na_2HPO_4 buffer, pH 6.0) and stopped by heat (95 °C, 5 min), followed by centrifugation and HPLC analysis. The glycan concentration (**2**) was 5 mM.

Preparative isolation of squarate monoamide esters

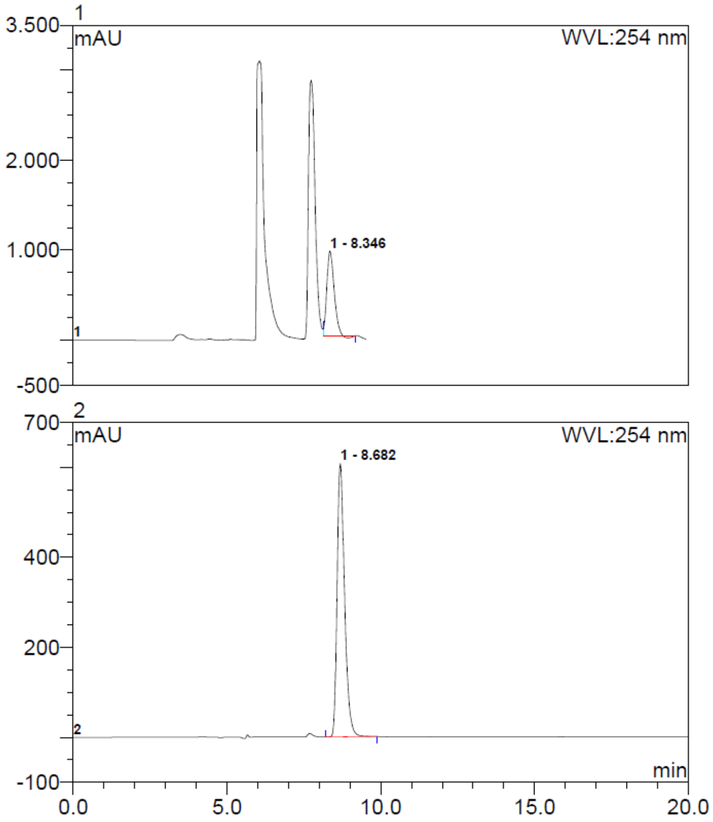

Figure S13. Isolation of squarate monamide ester **12** by preparative HPLC.

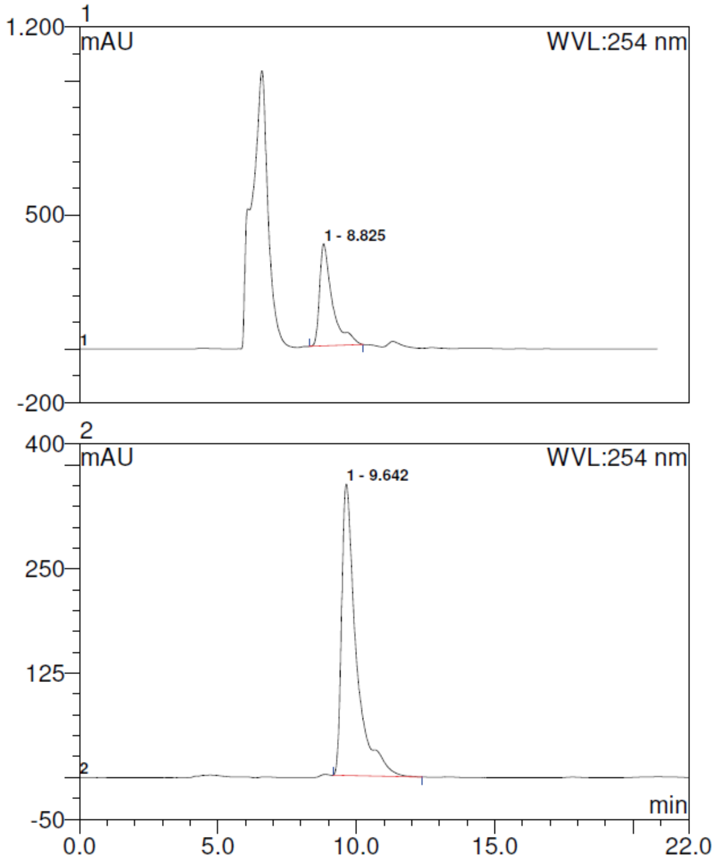

Figure S14. Isolation of squarate monamide ester **13** by preparative HPLC.

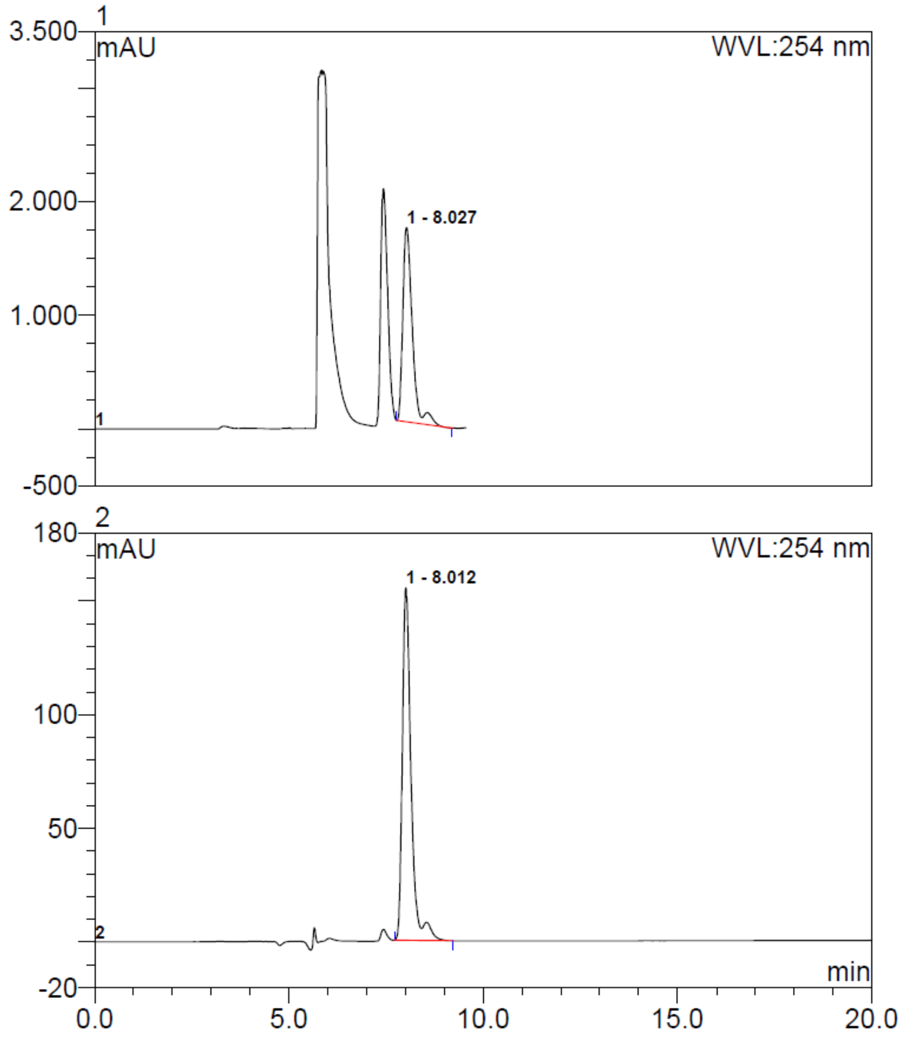

Figure S15. Isolation of squarate monamide ester **14** by preparative HPLC.

Table S1. Quantification and MS analysis of yielded squarate monoamide esters **12-14**

compound	amount [µmol]	molar yield [%]	calculated [M-H]⁻ m/z	observed [M-H]⁻ m/z	calculated [M-2H]²⁻ m/z	observed [M-2H]²⁻ m/z
**12**	1.48	67.2	972.2	972.5	485.7	486.0
**13**	1.74	87.5	972.2	972.6	485.7	485.8
**14**	1.90	74.5	972.2	972.5	485.7	485.8

Galectin binding assays

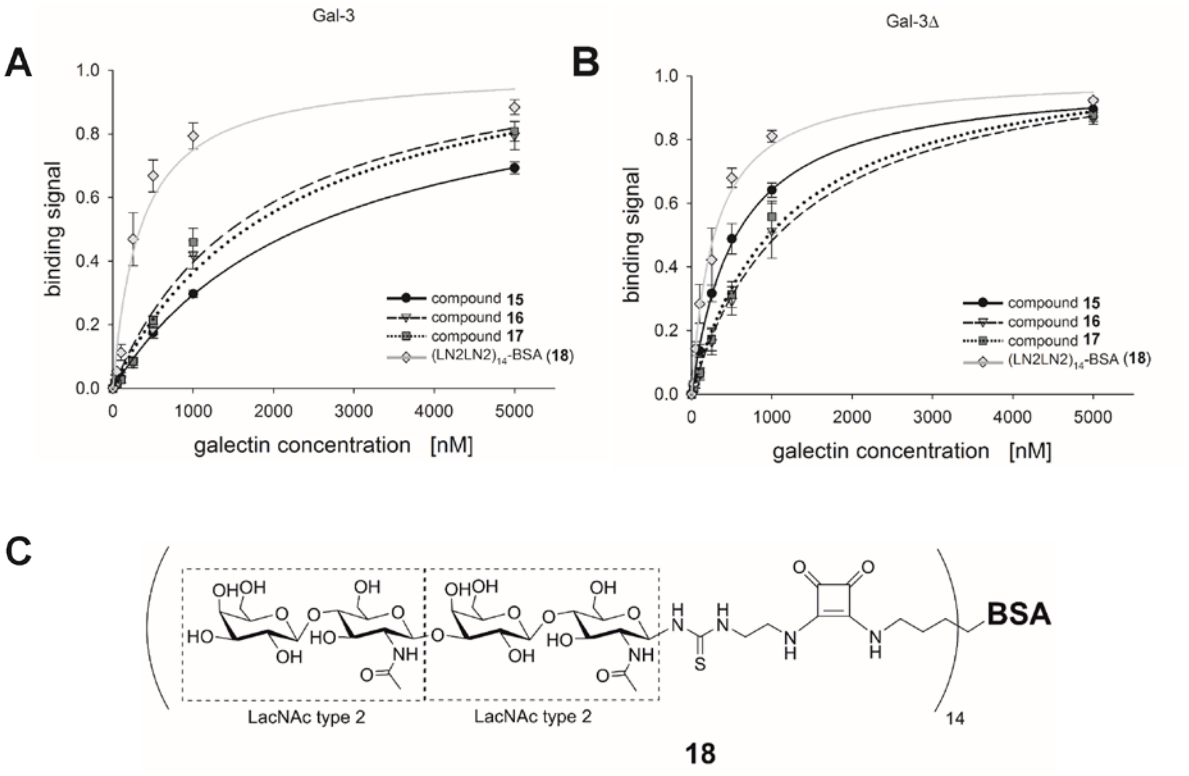

Figure S16. Multivalent neo-glycoproteins **15**-**17** and a LacNAc type 2 providing neo-glycoprotein ($(LN2LN2)_{14}$-BSA, **18**) [4] as ligands for Gal-3 (**A**) and Gal-3Δ (**B**). The structural composition of compound **18** is shown in **C**.

Mass spectrometry

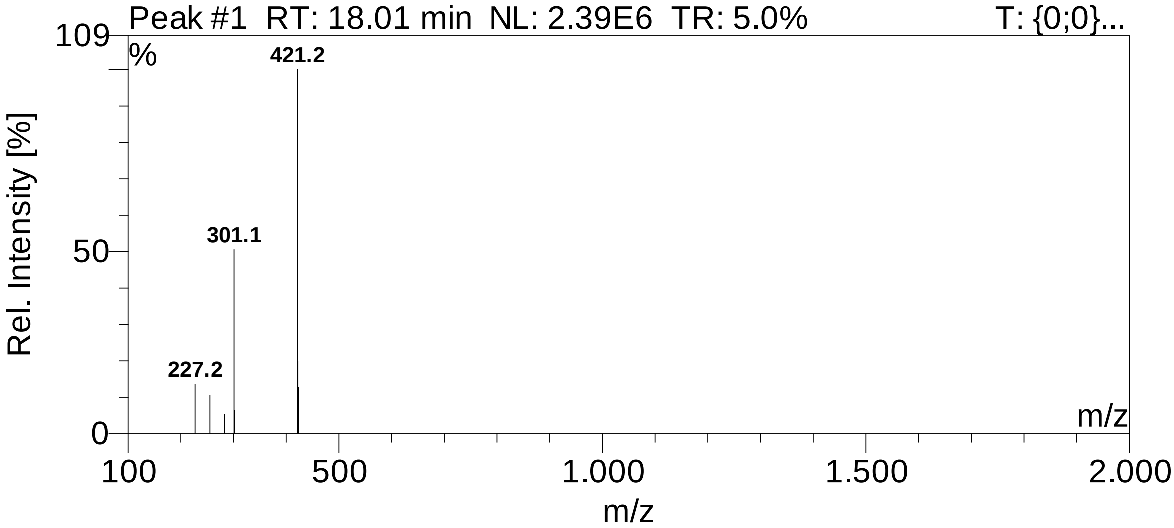

Figure MS1. Mass spectrum (ESI-) of GlcNAc-*t*Boc **1** ([M-H]$^-$, *m/z* 421.2).

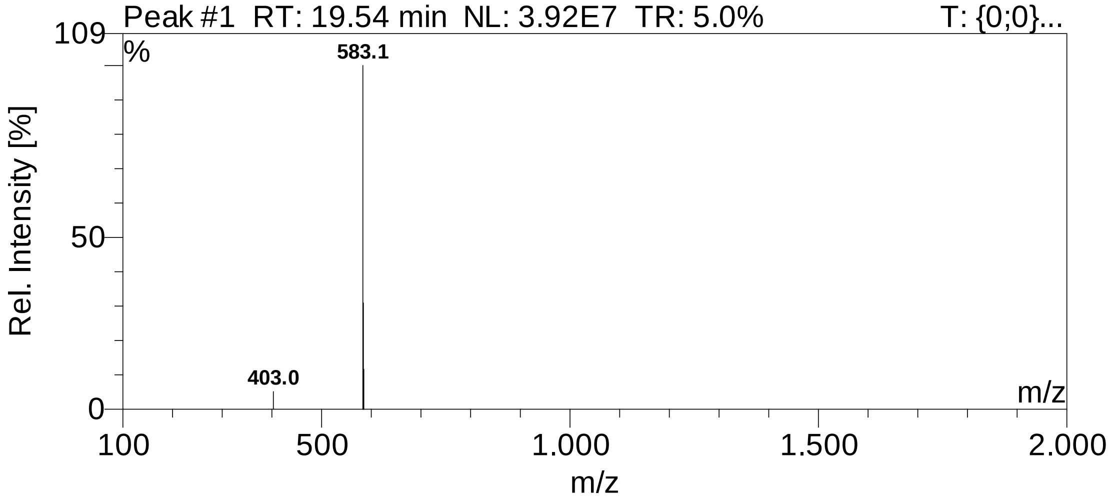

Figure MS2. Mass spectrum (ESI-) of LacNAc-*t*Boc **2** ([M-H]$^-$, *m/z* 583.1).

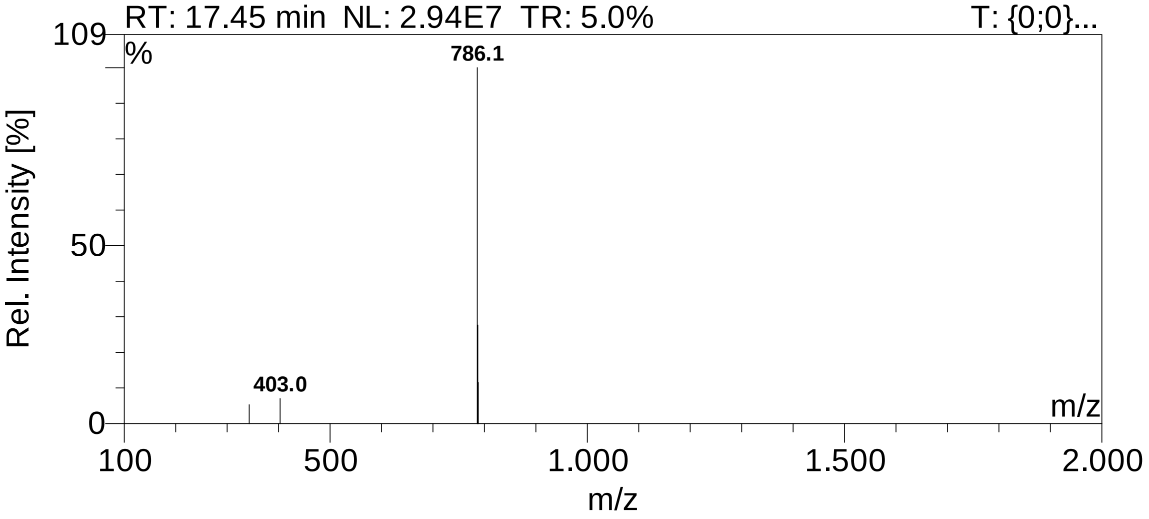

Figure MS3. Mass spectrum (ESI-) of GlcNAc-LacNAc-*t*Boc **3** ($[M-H]^-$, *m/z* 786.1).

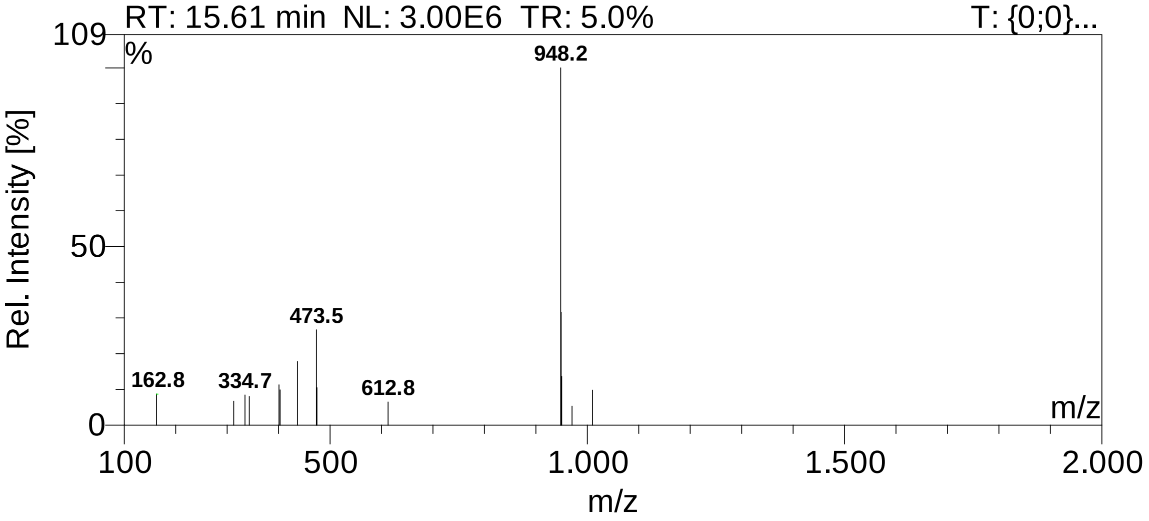

Figure MS4. Mass spectrum (ESI-) of $(LacNAc)_2$-*t*Boc **4** ($[M-H]^-$, *m/z* 948.2).

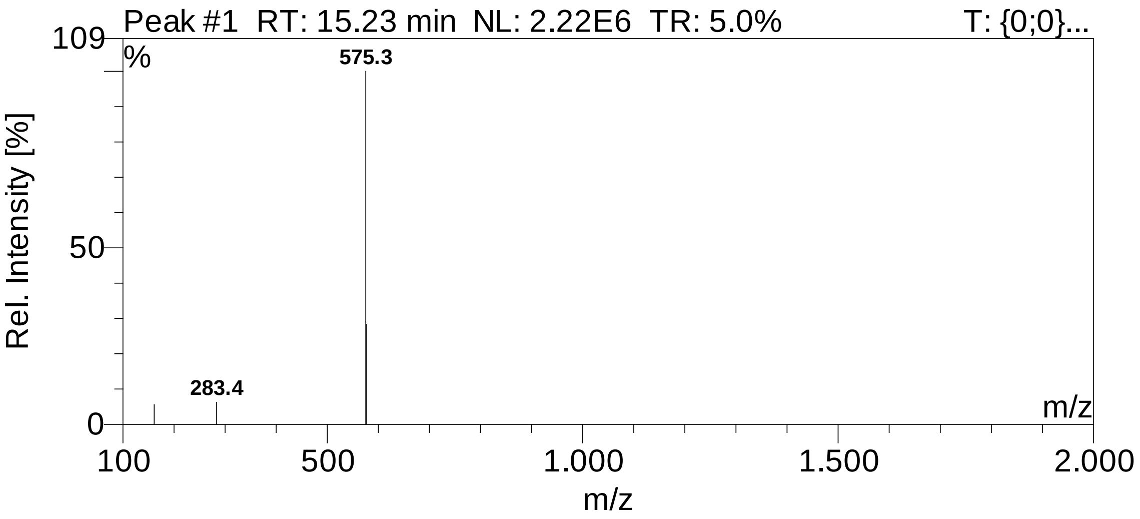

Figure MS5. Mass spectrum (ESI-) of GlcNAc-(LacNAc)$_2$-*t*Boc **5** ([M-2H]$^{2-}$, *m*/*z* 575.3).

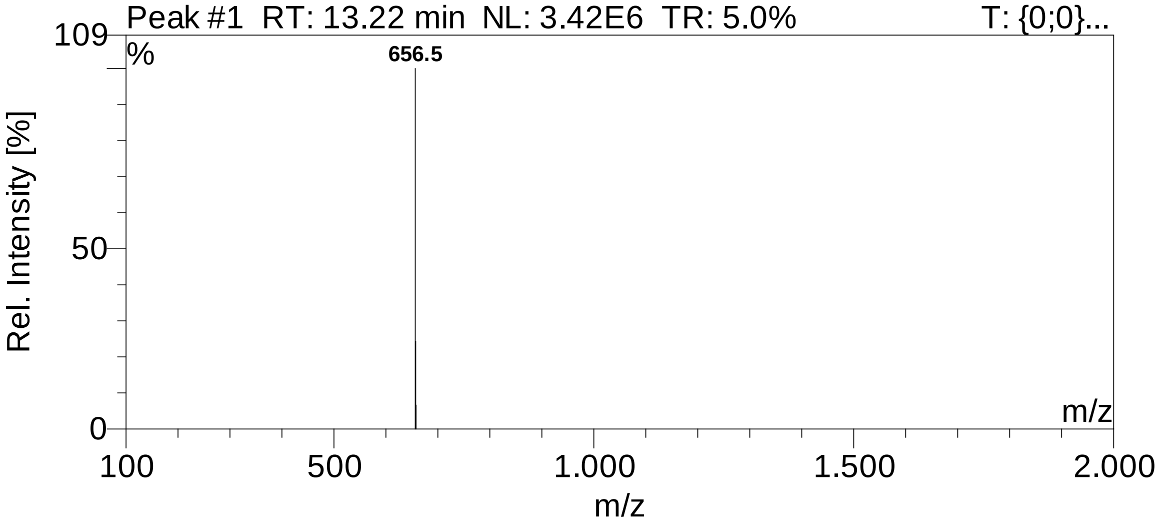

Figure MS6. Mass spectrum (ESI-) of (LacNAc)$_3$-*t*Boc **6** ([M-2H]$^{2-}$, *m*/*z* 656.5).

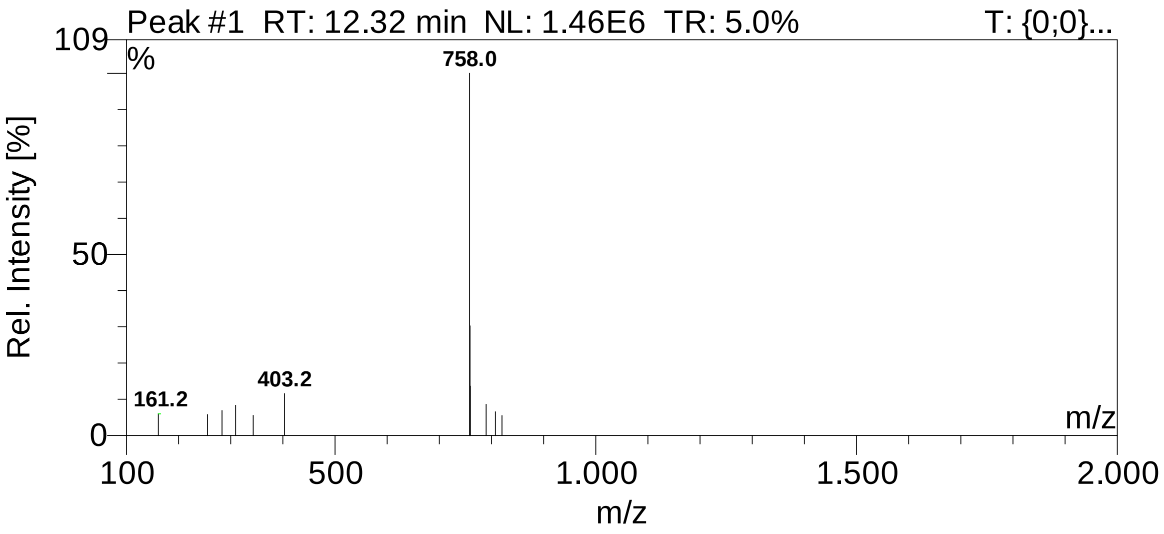

Figure MS7. Mass spectrum (ESI-) of GlcNAc-(LacNAc)$_3$-*t*Boc **7** ([M-2H]$^{2-}$, *m/z* 758.0).

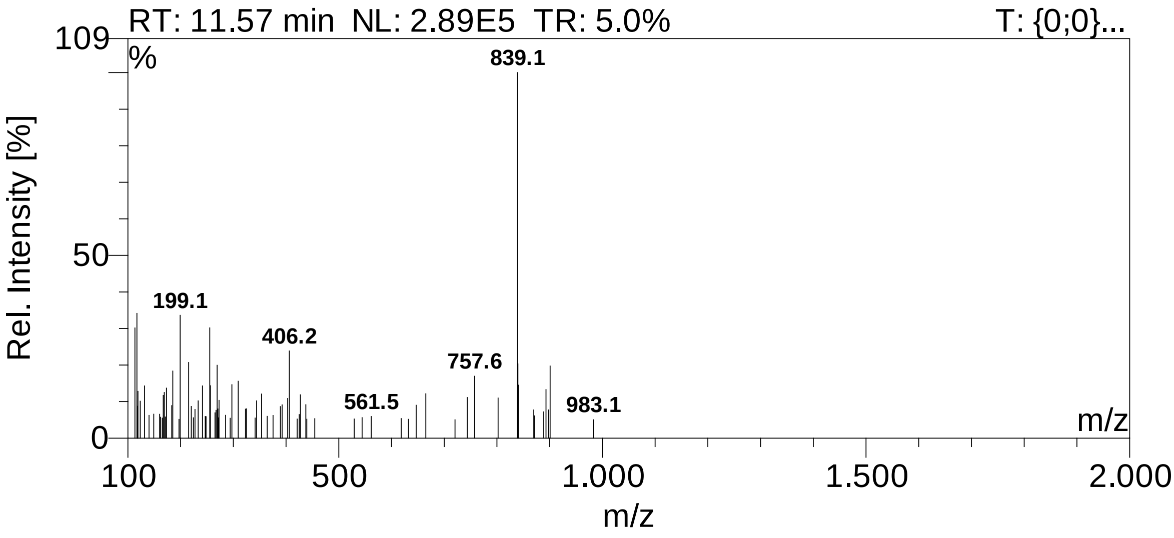

Figure MS8. Mass spectrum (ESI-) of (LacNAc)$_4$-*t*Boc **8** ([M-2H]$^{2-}$, *m/z* 839.1).

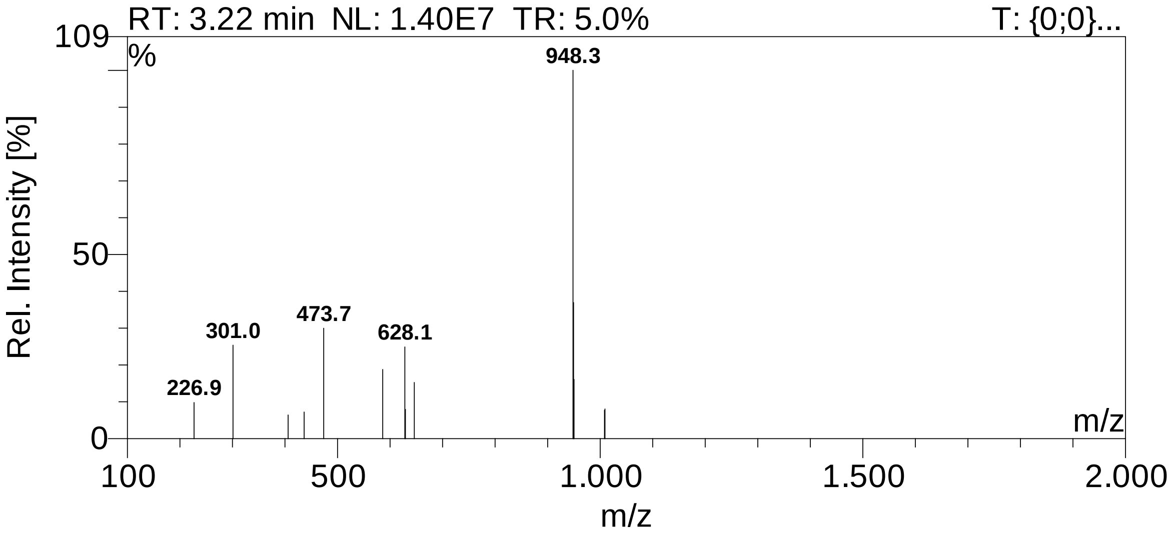

Figure MS9. Mass spectrum (ESI-) of LacNAc(type 2)-LacNAc(type 1)-*t*Boc **9** ($[M-2H]^{2-}$, *m/z* 948.3).

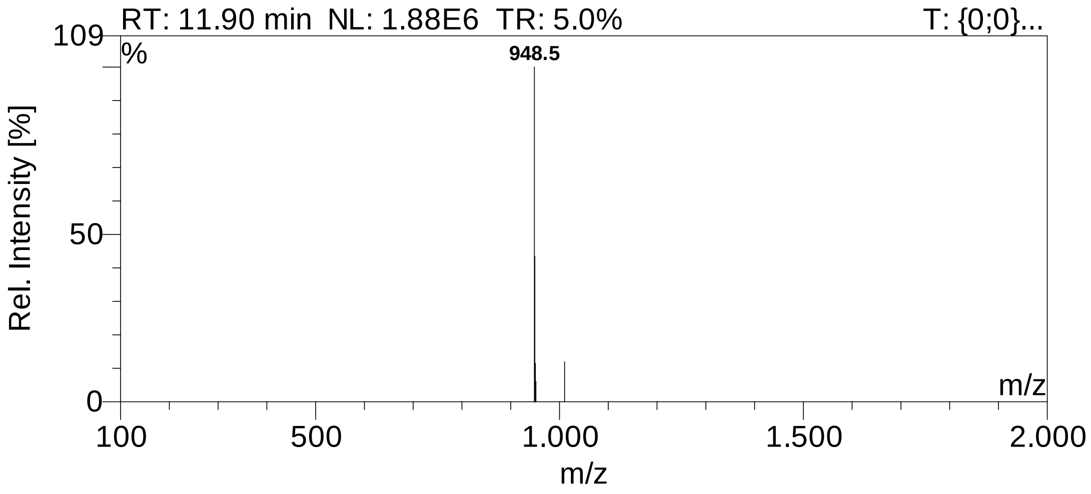

Figure MS10. Mass spectrum (ESI-) of LacNAc(type 1)-LacNAc(type 2)-*t*Boc **11** ($[M-2H]^{2-}$, *m/z* 948.5).

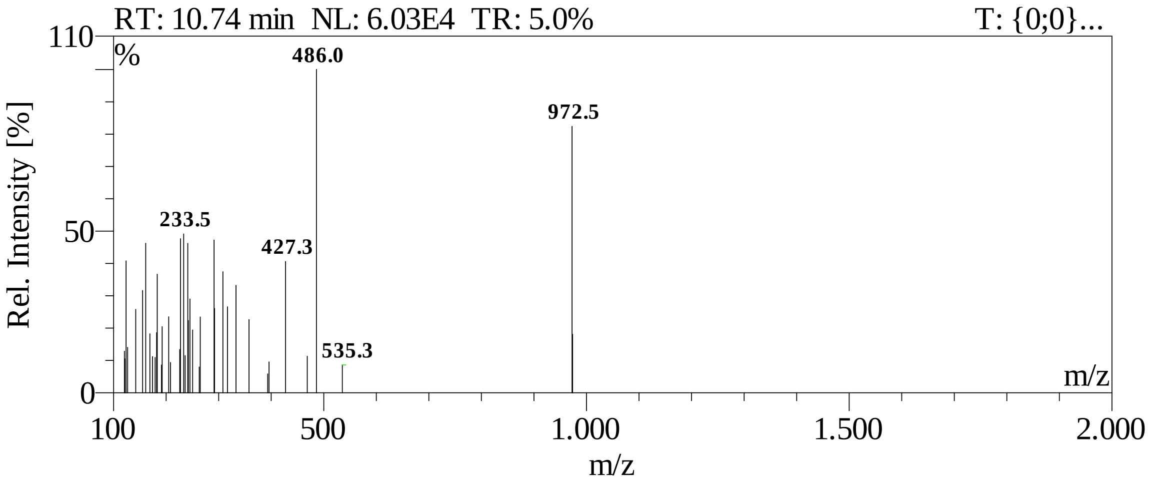

Figure MS11. Compound **12** ([M-H]$^-$, 972.5 *m*/*z*, [M-2H]$^{2-}$, 486.0 *m*/*z*).

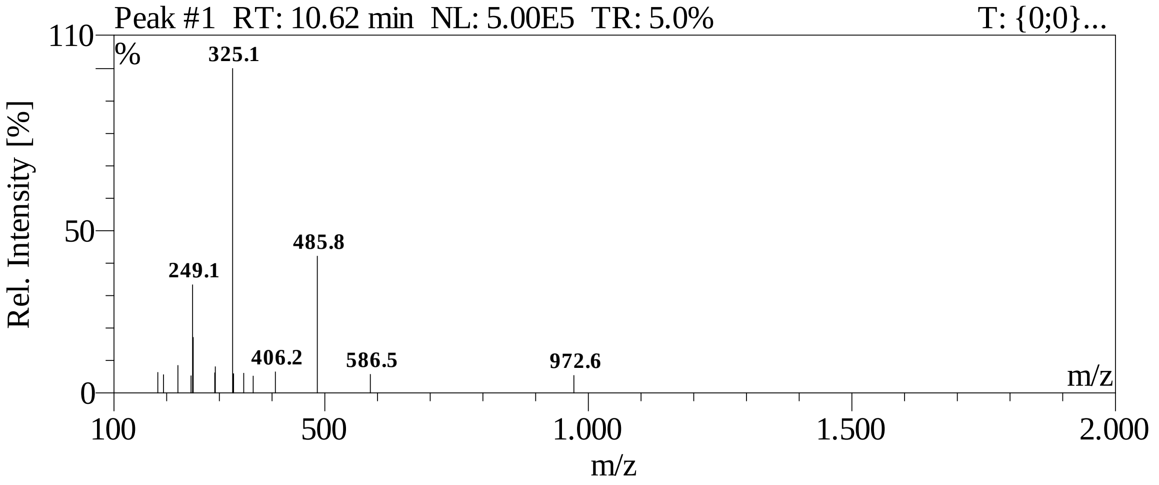

Figure MS12. Compound **13** ([M-H]$^-$, 972.6 *m*/*z*, [M-2H]$^{2-}$, 485.8 *m*/*z*).

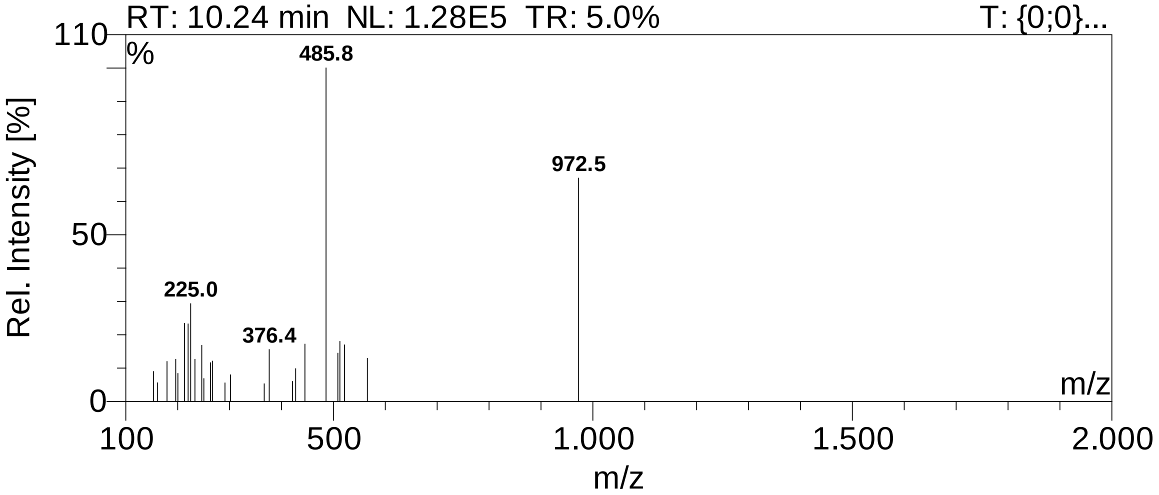

Figure MS13. Compound **14** ([M-H]$^-$, 792.5 *m*/*z*, [M-2H]$^{2-}$, 485.8 *m*/*z*).

References

1. Wahl, C.; Hirtz, D.; Elling, L. Multiplexed capillary electrophoresis as analytical tool for fast optimization of multi-enzyme cascade reactions – synthesis of nucleotide sugars. *Biotechnol. J.* **2016**, *11*, 1298-1308.
2. Henze, M.; You, D.-J.; Kamerke, C.; Hoffmann, N.; Angkawidjaja, C.; Ernst, S.; Pietruszka, J.; Kanaya, S.; Elling, L. Rational design of a glycosynthase by the crystal structure of β-galactosidase from bacillus circulans (bgac) and its use for the synthesis of n-acetyllactosamine type 1 glycan structures. *J. Biotechnol.* **2014**, *191*, 78-85.
3. Kamerke, C.; Pattky, M.; Huhn, C.; Elling, L. Synthesis of nucleotide-activated disaccharides with recombinant β3-galactosidase c from bacillus circulans. *J. Mol. Catal. B: Enzym.* **2013**, *89*, 73-81.
4. Böcker, S.; Laaf, D.; Elling, L. Galectin binding to neo-glycoproteins: Lacdinac conjugated bsa as ligand for human galectin-3. *Biomolecules* **2015**, *5*, 1671-1696.
